# KIF1A is kinetically tuned to be a superengaging motor under hindering loads

**DOI:** 10.1073/pnas.2216903120

**Published:** 2023-01-04

**Authors:** Serapion Pyrpassopoulos, Allison M. Gicking, Taylor M. Zaniewski, William O. Hancock, E. Michael Ostap

**Affiliations:** ^a^The Pennsylvania Muscle Institute, Department of Physiology, Perelman School of Medicine, University of Pennsylvania, Philadelphia, PA 19104; ^b^Center for Engineering Mechanobiology, Perelman School of Medicine, University of Pennsylvania, Philadelphia, PA 19104; ^c^Department of Biomedical Engineering, Pennsylvania State University, University Park, PA 16802; ^d^Department of Chemistry, Pennsylvania State University, University Park, PA 16802

**Keywords:** kinesin, single-molecule, optical tweezers

## Abstract

KIF1A is a highly processive vesicle transport motor in the kinesin-3 family. Mutations in KIF1A lead to neurodegenerative diseases including hereditary spastic paraplegia. We applied optical tweezers to study the ability of KIF1A to generate and sustain force against hindering loads. We used both the three-bead assay, where force is oriented parallel to the microtubule, and the traditional single-bead assay, where force is directed along the radius of the bead, resulting in a vertical force component. The average force and attachment duration of KIF1A in the three-bead assay were substantially greater than those observed in the single-bead assay. Thus, vertical forces accelerate termination of force ramps of KIF1A. Average KIF1A termination forces were slightly lower than the kinesin-1 KIF5B, and the median attachment duration of KIF1A was >10-fold shorter than KIF5B under hindering loads. KIF1A rapidly reengages with microtubules after detachment, as observed previously. Strikingly, quantification enabled by the three-bead assay shows that reengagement largely occurs within 2 ms of detachment, indicating that KIF1A has a nearly 10-fold faster reengagement rate than KIF5B. We found that rapid microtubule reengagement is not due to KIF1A’s positively charged loop-12; however, removal of charge from this loop diminished the unloaded run length at near physiological ionic strength. Both loop-12 and the microtubule nucleotide state have modulatory effects on reengagement under load, suggesting a role for the microtubule lattice in KIF1A reengagement. Our results reveal adaptations of KIF1A that lead to a model of superengaging transport under load.

## Significance

KIF1A is a superprocessive cytoskeletal motor that transports intracellular cargo in neurons and is a target of mutations linked to neurodegenerative diseases. In this work we report that KIF1A has evolved kinetic features that allow it to remain dynamically engaged with microtubule in the presence of hindering load. Once detached by load, KIF1A can reattach to a new region on the microtubule within milliseconds to continue transport. Quantification of this behavior was made possible by implementing an assay geometry, which minimizes loads that force the motor away from the microtubule. These findings reveal kinetic parameters not yet described within the kinesin superfamily that result in a motor with mechanochemistry that facilitates bidirectional transport and the ability to navigate around obstacles.

**Fig. 1.**
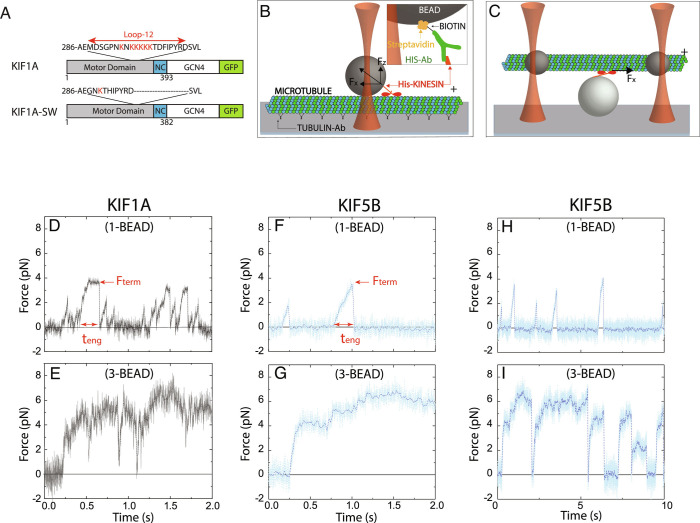
KIF1A performance in single-bead and three-bead assays. (*A*) KIF1A constructs used in this study. Wild-type KIF1A consists of the motor domain the neck coil region (NC) of rat KIF1A, followed by a leucine zipper dimerization domain (GCN4) and a C-terminal GFP and His_6_-tag. In the KIF1A-SW swap mutant, the loop-12 was replaced by the corresponding sequence of *Drosophila* kinesin-1. (*B* and *C*): Diagram of the (*B*) single-bead and (*C*) three-bead assays with attachment strategy (*Inset*); beads are not drawn to scale. (*D* and *E*): Representative force traces of KIF1A at 2 mM ATP in (*D*) the single-bead assay and (*E*) the three-bead assay [for a time expanded version of panels (*D*) and (*E*), see *SI Appendix*, Fig. S2]. F_term_ is defined as the force at the termination of a force ramp and t_eng_ is defined as the duration of a force ramp. (*F* and *G*): Force traces of KIF5B using (*F*) the single-bead assay and (*G*) the three-bead assay with the same time axis as *D* and *E*. (*H* and *I*): KIF5B force traces at expanded time scale, showing larger intervals between force ramps and long force plateaus in the three-bead geometry. Additional force traces are presented in *SI Appendix*, Fig. S3.

KIF1A is a cytoskeletal motor in the kinesin-3 family that transports intracellular cargo in axons and dendrites (1, 2). A number of human mutations in KIF1A have been identified that lead to neurodegenerative diseases, termed KIF1A-associated neurological disorders (KAND) (3, 4). KIF1A is functionally distinctive in the kinesin superfamily in that it has a fast-stepping rate and enhanced processivity in the absence of mechanical loads compared to other characterized motors. A positively charged loop-12 insert, the “K-loop,” (Fig. 1*A*) is unique to the kinesin-3 family and has been linked to the superprocessive behavior of KIF1A monomers (5, 6). However from single-molecule experiments of dimeric KIF1A, there are disagreements regarding the contribution of the K-loop to the superprocessivity of KIF1A dimers (7–11). Despite being superprocessive in the absence of load, previous work has shown that mechanical loads that resist plus end-directed stepping cause KIF1A to detach from the microtubule more readily than the well-studied kinesin-1 (12–15).

A recent biochemical study exploring the mechanochemical adaptations of KIF1A suggested that rear-head detachment is an order of magnitude faster than found for kinesins-1 or -2, and that this feature helps to explain its rapid stepping rate (16). This kinetic feature also results in a predominant steady-state intermediate that is bound via a single “weakly-bound” posthydrolysis motor domain through electrostatic interactions with the microtubule. This single-head microtubule interaction may result in a molecule that is vulnerable to detachment under mechanical load. Indeed, a recent study using a single-bead optical tweezer found that KIF1A bound for relatively short durations under load and generated stall forces of 3.1 pN, compared with 4.6 pN for kinesin-1 (15). Interestingly, KIF1A was found to recover processive stepping after detachment more readily than kinesin-1, and this property has been attributed to the unique K-loop (8, 16).

The specific sequences and biochemical tuning that underlie the superprocessivity and force sensitivity of KIF1A are still under investigation. The α4-helix, which forms a substantial part of the microtubule-binding interface, is conserved between the kinesin-1 and kinesin-3 families, but there are positively charged residues in loop-8, loop-11, and the α6-helix of KIF1A that, when substituted for their kinesin-1 counterparts, reduce the unloaded run length substantially (17). Furthermore, the N-terminal cover strand of kinesin-3, which stabilizes the docked neck linker and contributes to force generation, is shorter than that of kinesin-1 and forms a less extensive hydrogen bonding network with the motor domain and neck linker (15, 18). These unique structural features within the catalytic core and at the motor–microtubule interface raise the possibility that KIF1A motor kinetics are affected by force differently than kinesin-1.

Given the unique connection between the neck-linker and motor of KIF1A, it is important to consider how the geometry of forces applied to the motor affects its mechanochemistry. The single-bead assay (Fig. 1*B*), which is commonly used for measuring the force generated by kinesin motors [including KIF1A (10, 15)], introduces a vertical component F_z_ to the force applied to kinesin due to contact of the bead with the underlying surface-immobilized microtubule (19, 20). This vertical force component, which is difficult to measure directly, acts to separate the motor from the microtubule. The impact of F_z_ can be significant; for instance, for a bead with a 520-nm diameter, F_z_ is estimated to be more than twice the magnitude of the horizontal force component, F_x_ that is measured by the laser trap (19, 20). Fortunately, the vertical force can be minimized using a three-bead assay (Fig. 1*C*), in which the motor is attached to a surface-immobilized bead and a microtubule “dumbbell” is held above it by two laser-trapped beads attached near the microtubule ends (20, 21). For instance, for a dumbbell with beads of 820 nm in diameter and a 10-µm microtubule, the vertical component is expected to be more than an order of magnitude smaller than the horizontal component (20). In recent work, it was found that the microtubule detachment rate of human kinesin-1, KIF5B, was substantially slower in the three-bead assay (20), suggesting that the vertical force inherent to the single-bead assay contributes to the measured motor detachment kinetics. Thus, given the recent finding that KIF1A detaches from microtubules more readily under force (15), it is important to examine the contribution of parallel and vertical forces to processive stepping.

In the present work, we investigated the performance of KIF1A in single- and three-bead optical trap assays and compared its performance with kinesin-1. We found that KIF1A can achieve forces up to 6 pN, and it terminates its processive runs faster than kinesin-1 under opposing loads. However, KIF1A is superengaging, in that after termination of its force ramps, the motor quickly reengages and initiates a new force ramp within 2 ms. We also found that at near physiological (184 mM) ionic strength, the K-loop contributes substantially to the unloaded run length, but only minimally to the load-dependent detachment kinetics. These results suggest that during transport of vesicles in axons and dendrites, where forces are expected to be predominantly oriented parallel to the microtubule, KIF1A can detach and rapidly recover motility under load, an adaptation that facilitates bidirectional transport and navigation around obstacles.

## Results

### KIF1A Generates Substantial Forces and Reengages More Frequently with the Microtubule than KIF5B.

To probe the force-generating properties of KIF1A, we used optical tweezers in both a single-bead and three-bead configuration (Fig. 1 *B* and *C*) at saturating adenosine triphosphate (ATP) (2 mM) in BRB80 buffer (pH 6.9, 184 mM ionic strength). Because full-length KIF1A molecules adopt an autoinhibited conformation (3, 22, 23), we used a *Rattus norvegicus* KIF1A construct consisting of the motor and neck coil domains dimerized by a GCN4 leucine zipper and followed by a green fluorescent protein (GFP) (7) (Fig. 1*A*). KIF1A concentrations used in the optical tweezer experiments were sufficiently low to ensure that observed interactions are due to single KIF1A dimers (see *Materials and Methods*).

In the single-bead assay, KIF1A molecules pulled the bead out of the center of the stationary optical trap to forces of ~ 4 pN (Fig. 1*D*). Terminations of force ramps were followed by strictly monotonic decreases in force as the bead relaxed back toward the center of the optical trap. By averaging many such events, we found that the relaxation time was ≥2 ms, which is near the expected relaxation time of a single bead in the absence of any interactions with the microtubule (*SI Appendix*, Fig. S1) (24). These rearward displacements may reflect complete dissociation of KIF1A from the microtubule termed here as detachment; alternatively, they could reflect KIF1A slipping backward while maintaining weak association with the microtubule, as shown previously for other kinesins (25–27). As we cannot differentiate between these attachment states, we refer to the force value at the termination of each force ramp as the termination force (F_term_; Fig. 1 *D* and *F*).

Following termination of the force ramp, KIF1A can quickly reengage with the surface-immobilized microtubule and resume forward motion (Fig. 1*D*). Successive KIF1A force ramps were more closely spaced in time than those measured for KIF5B under identical assay conditions. The maximal KIF1A termination forces (F_term_) of ~4 pN were lower than KIF5B, and the duration of the force ramps, defined as the engagement time (t_eng_), were shorter for KIF1A than for KIF5B (Fig. 1 *D* and *F*). The lower forces and rapid reengagement kinetics of KIF1A agree with a recent single-bead optical trapping study using a rat KIF1A construct (15).

In the single-bead assay, forces are applied to kinesin in directions both parallel and normal to the long-axis of the microtubule [Fig. 1*B*; (19, 20)]. To investigate the force-generating properties of KIF1A in the absence of this normal force component, we used the three-bead assay, in which the motor is attached to a surface-immobilized bead and a microtubule “dumbbell” is held above it by two laser-trapped beads attached near the microtubule ends (Fig. 1*C* and *Materials and Methods*). In the three-bead assay, KIF1A developed maximal forces of ~6 pN, substantially larger than in the single-bead assay and close to the forces generated by KIF5B (see *Results* below). Notably, the durations of the KIF1A force ramps were still substantially shorter than observed for KIF5B (Fig. 1 *E* and *G*). After termination of a force ramp, KIF1A rapidly reengaged and initiated the next force ramp before the dumbbell fully relaxed to the zero-force baseline. This rapid reengagement was rarely observed for KIF5B (Fig. 1 *G* and *I*).

### In the Absence of Vertical Forces KIF1A Generates Large Pulling Forces and Repetitively Reengages with the Microtubule.

**Fig. 2.**
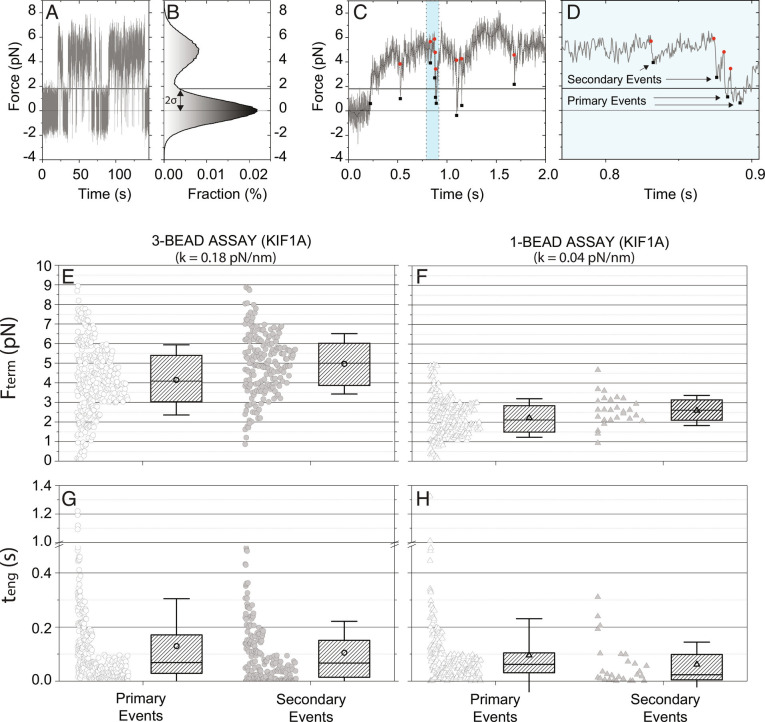
Quantification of KIF1A primary and secondary force ramps. (*A*) Long duration force trace of KIF1A in the three-bead assay. (*B*) Corresponding stationary distribution of force, exhibiting a peak at the zero-force equilibrium position and a ramp force peak at 5 pN. Primary events are defined as force ramps that start within two-SDs of the zero-force baseline (horizontal 2σ line at F = 1.8 pN). (*C*) Sample force trace in the three-bead assay showing multiple force ramp termination events (red dots) and reengagement events (black squares). Reengagements that initiate below the 2σ line are considered primary binding events, whereas events that start above the threshold are considered secondary events. (*D*) Expanded view of highlighted portion of (*C*). (*E* and *F*): KIF1A termination forces, F_term_ for primary and secondary events in the (*E*) three-bead and (*F*) single-bead assays. In each case, raw data are shown at *Left* and the average (open circle and triangle), median (horizontal line), quartile (boxes), and SD (error bars) shown at *Right*. (*G* and *H*): Engagement durations, t_eng_ for primary and secondary events in the (*G*) single-bead and (*H*) three-bead assay. Raw data are shown at *Left* and the mean (open circles and triangles), quartile (shaded box), and SD (error bars) are shown at *Right*. For the values of <F_term_> and median-t_eng_ see *SI Appendix*, Table S1.

To isolate the influences of vertical and horizontal forces on KIF1A stepping, we quantified the force-generating capacity of KIF1A and the microtubule reengagement kinetics following termination of a force ramp. A representative example of a long (>100 s) trace that contains many consecutive KIF1A force ramps is shown in Fig. 2*A*. Since the frequency of reengagement events can be affected by the vertical distance between the kinesin and microtubule (20, 28), in both the single- and three-bead assays, the separation between kinesin and microtubule was decreased by ~20 nm every 20 s, until single-molecule interactions were observed. When the distribution of instantaneous forces was plotted (Fig. 2*B*), two clear modes were apparent: a peak around the zero-force baseline and a peak around the average force where force ramps terminated. For our analysis, force ramps that initiated at forces within two-SDs of the zero-force baseline were termed primary events, whereas force ramps that initiated at forces greater than two-SDs from the baseline were termed secondary events (Fig. 2 *C* and *D*). For KIF1A, 39% of the force ramps in the three-bead assay qualified as secondary events, whereas only 11% qualified as secondary events in the single-bead assay (*SI Appendix*, Table S1). The difference in the number of secondary events between the assays may result from the microtubule remaining near the immobilized motor in the three-bead trap, whereas the motor position is less constrained in the single-bead trap due to potential rotation of the bead. It is also possible that tensile forces applied by the two traps on the microtubule in the three-bead assay (*Materials and Methods*) may deform the microtubule lattice and thereby enhance motor reengagement kinetics (29).

To characterize the mechanical performance of KIF1A against hindering loads, we quantified the distributions of termination forces, F_term_, and the durations that motors engaged with microtubules before termination of a force ramp, t_eng_. For both the single-bead and three-bead assays, <F_term_> was slightly higher for the secondary events relative to the primary ones (Fig. 2 *E* and *F* and *SI Appendix*, Table S2). The higher forces may be expected, because primary events begin at lower initial forces, and hence require more time to build to higher forces; it also reflects the fact that force ramps usually terminate before the motor reaches a stable force. To better quantify the kinetics of this termination process, we compared the distribution of motor engagement times, t_eng_, in the three-bead assay (Fig. 2 *G* and *H*; values in *SI Appendix*, Table S2). The median t_eng_ was similar for primary and secondary events, at 69 ms and 67 ms, respectively, consistent with primary and secondary events reflecting similar motor engagement processes and differing only in their initial starting positions. In the single-bead assay, the primary events had similar median durations as the three bead (62 ms), whereas the secondary events were fairly rare and had shorter median duration (23 ms).

Because the microtubule dumbbell was pretensioned to reduce thermal noise, higher trap stiffnesses were used with the three-bead assay. The higher stiffness also compensated for the larger viscous drag of the dumbbell compared with a single bead, resulting in the relaxation times being similar for the two assays (*SI Appendix*, Fig. S1). As a result of this higher stiffness, the loading rate during force ramps (dF_x_/dt =  k_x_·v_x_) was faster in the three-bead assay, raising the possibility that the lower termination forces in the single-bead assay may result from the longer time required to generate high forces. To rule out this possibility, we repeated the single-bead assay at a higher trap stiffness and found that, although F_term_ increased slightly, it was still substantially lower than the value for the three-bead assay (*SI Appendix*, Fig. S4*A*). Furthermore, using a more comparable trap stiffness in the single-bead assay, the median engagement time fell to 35 ms, highlighting shorter engagement times in the single-bead assay (*SI Appendix*, Fig. S4*B*). Therefore, termination forces were higher in the three-bead assay than in the single-bead assay, consistent with the vertical forces inherent in the single-bead assay limiting the duration of the force ramps.

### KIF1A Engagement Times Are Short under Load and Reengagement Occurs within Milliseconds.

**Fig. 3.**
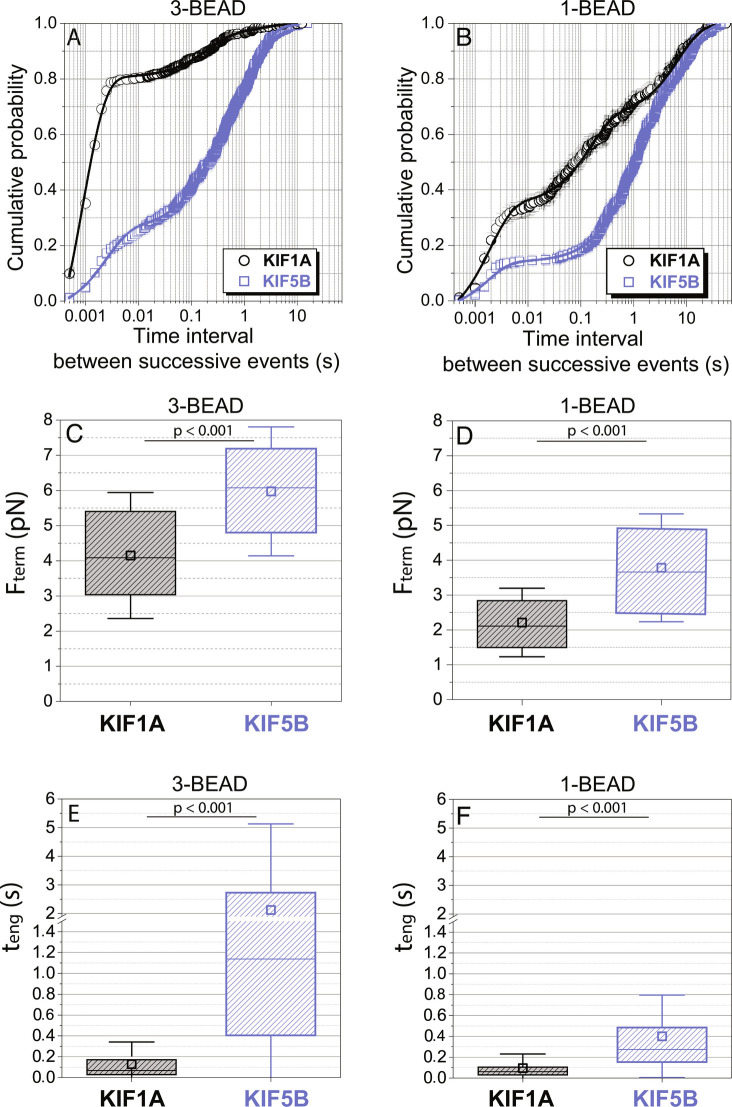
Comparison of KIF1A and KIF5B engagement kinetics and termination forces. (*A* and *B*): Cumulative probability distribution of time intervals between successive force ramps (t_restart_) for KIF1A and KIF5B in the (*A*) three-bead and (*B*) single-bead assays. Data include both primary and secondary events. Error bars are calculated using the bootstrap method (30), and solid lines represent fitting to a three-exponential function with offset (see *Materials and Methods*). (*C* and *D*): Comparison of KIF1A and KIF5B termination forces, F_term_ for primary events in the (*C*) three-bead and (*D*) single-bead assays. Data are presented as mean (open squares), median (horizontal line), quartiles (shaded boxes), and SD (error bars). (*E* and *F*): Comparison of KIF1A and KIF5B engagement durations, t_eng_ for primary events in the (*E*) three-bead and (*F*) single-bead assays. Note the break introduced at 1.5 s in the Y-axis due the large difference between the median values of t_eng_ between KIF1A (0.069 s) and KIF5B (1.1 s) in the three-bead assay. Statistical comparisons were done by applying the Mann–Whitney test.

A consistent feature of KIF1A behavior in both the single- and three-bead assays was the rapid reengagement of the motor with the microtubule following the termination of a force ramp. This behavior was observed previously in a single-bead study (15), but not quantified. To characterize this reengagement behavior, we determined the restart time, t_restart_, defined as the time between termination of one force ramp and initiation of the next. For KIF1A and KIF5B in the two optical trapping geometries, the cumulative probability distribution of restart times showed a population of fast restart events on the ms timescale and two slower populations with time constants >100 ms (Fig. 3 *A* and *B*). The cumulative distributions of t_restart_ were fitted to the sum of three exponentially distributed populations. In the three-bead assay, the time constant of the fastest phase was 0.89 ms for KIF1A and 2.5 ms for KIF5B (Table 1), which are on the order of the dead time of the experiment set by the relaxation time of the trapped beads (see *Methods*). Strikingly, 79% of KIF1A reengagement events occur within the fast phase, compared with only 25% of KIF5B reengagements (Table 1). For both motors, the amplitude of the fast phase is smaller for the single-bead assay (Table 1), suggesting that the assay geometry significantly impacts the reengagement times (Fig. 3*B*). The slower phases are likely due to motors detaching from the microtubule with reengagement being limited by the steric constraints of the experimental geometry and the motor kinetics.

**Table 1.** Results of fitting cumulative probability of reengagement to a triexponential function with relative amplitudes A and time constants τ.(^*^)

	A1 (%)	τ_1_(s)	A2 (%)	τ_2_(s)	A3 (%)	τ_3_(s)
KIF1A-1B	34 ± 1.4	0.0019 ± 0.00022	32 ± 1.3	0.15 ± 0.015	34 ± 1.0	7.2 ± 0.43
KIF1A-3B	79 ± 1.9	0.00089 ± 0.000040	17 ± 0.90	0.20 ± 0.019	5.0 ± 0.82	2.0 ± 0.53
KIF5B-1B	14 ± 0.46	0.0017 ± 0.0020	50 ± 1.2	0.96 ± 0.033	37 ± 1.0	9.3 ± 0.37
KIF5B-3B	25 ± 0.68	0.0025 ± 0.00019	26 ± 0.43	0.17 ± 0.012	49 ± 1.1	1.3 ± 0.030
KIF1A-SW-3B	66 ± 1.9	0.0011 ± 0.000065	14 ± 1.1	0.14 ± 0.020	19 ± 1.1	1.2 ± 0.084

^*^The triexponential function is given in *Materials and Methods*. The relative amplitudes A_i_, the characteristic times τ_I,_ and the errors are calculated from the 95% CIs of each fitting parameter (see *Materials and Methods*). Error-weighted fits were performed using bootstrap errors (30).

To compare the ability of the two motors to generate and sustain forces against hindering loads oriented exclusively parallel to the microtubule, we compared the average F_term_ and median t_eng_ in the three-bead assay. As shown in Fig. 3*C*, <F_term_> was ~6 pN for KIF5B, but was only ~4 pN for KIF1A. This reduced capacity of KIF1A to generate and sustain forces was also seen in the single-bead assay (Fig. 3*D*). Therefore, even though KIF5B steps at less than half the speed of KIF1A and thus takes longer time to generate large forces, the KIF5B force ramps terminate at higher forces. Consistent with these higher forces, the median engagement time during force ramps in the three-bead assay was more than an order of magnitude shorter for KIF1A than for KIF5B (Fig. 3*E*), and shorter engagement times for KIF1A were also seen in the single-bead assay (Fig. 3*F*). In summary, KIF1A force ramps terminate more readily than KIF5B, and consequently only ~15% of KIF1A ramps reach 6 pN, whereas ~50% of KIF5B ramps reach and exceed 6 pN (*SI Appendix*, Fig. S5).

### The KIF1A Loop-12 Contributes to Superprocessivity but Does Not Enhance Initial Landing on Microtubules.

**Fig. 4.**
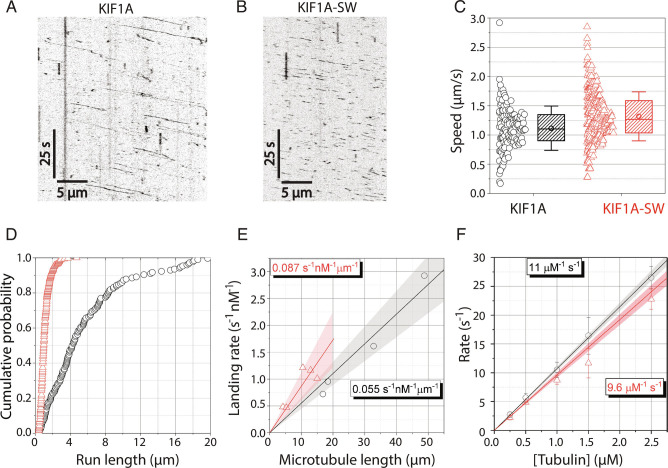
Influence of loop-12 on KIF1A performance under zero load. (*A* and *B*) Kymographs from single-molecule TIRF assays of (*A*) KIF1A and (*B*) KIF1A-SW on control microtubules in 2 mM ATP. (*C*) Comparison of single-molecule speeds between KIF1A (black circles) and KIF1A-SW (red triangles), showing raw data at *Left* and mean (open symbols), median (horizontal line), quartile (shaded box), and SD (error bar) at right. (*D*) Cumulative probability distribution of KIF1A and KIF1A-SW run lengths, using the same symbols and colors as in (*C*). (*E*) Plot of the single-molecule landing rate (s^−1^·nM^−1^) for KIF1A (black circles) and KIF1A-SW (red triangles) as a function of the microtubule length (μm). The solid lines are linear fits, which correspond to the landing rates (s^−1^·nM^−1^·μm^−1^) for each motor. Shaded areas represent the 95% confidence bands of the linear fits. (*F*) Plot of the mantADP release rate upon mixing mantADP bound motors with different concentrations of taxol-stabilized microtubules by stopped-flow. The linear fits to the data represent the bimolecular on-rate for KIF1A binding to microtubules (μM^−1^·s^−1^). Shaded areas represent the 95% confidence bands of the linear fits.

A distinctive feature of the KIF1A motor domain is a loop-12 insert containing six positively charged lysines that are thought to interact electrostatically with the negatively charged C-terminal tails of tubulin (5, 6) (Fig. 1*A*). To test the contribution of loop-12 to the microtubule engagement duration and superprocessivity of KIF1A at near physiological ionic strength, we made a loop swap mutant, KIF1A-SW, by exchanging the native KIF1A loop-12 that contains the six lysines for loop-12 from *Drosophila* kinesin-1, which contains only one lysine (Fig. 1*A*). Single-molecule TIRF experiments in BRB80 buffer showed that in the absence of external forces, KIF1A and KIF1A-SW move along surface immobilized microtubules at similar average speeds <V> of 1.2 ± 0.36 μm/s and 1.3 ± 0.42 μm/s, respectively (Fig. 4 *A–C* and *SI Appendix*, Table S3). Notably, the average run length <RL> of KIF1A-SW (1.1 ± 0.56 μm) was approximately sixfold lower than for KIF1A (6.3 ± 4.2 μm) (Fig. 4*D* and *SI Appendix*, Table S3). To ensure that the observed run lengths are due to single molecules and not multimeric KIF1A aggregates, we quantified the fluorescence intensity distribution from our single-molecule events and its correlation with the run length. The intensity distribution was unimodal, and there was no correlation between fluorescence intensity and run length indicating that our results reflect single-molecule behavior (*SI Appendix*, Fig. S6).

Previous studies that used a loop-swap construct found that swapping the lysine containing region of loop-12 of KIF1A had a minimal effect on the run length at low-ionic strength (BRB12 buffer, pH 6.9 and 36 mM ionic strength; see *SI Appendix*), although it did enhance the KIF1A landing rate (8). To resolve this discrepancy, we carried out single-molecule experiments in BRB12 buffer and found that the run lengths of KIF1A and KIF1A-SW were similar to one another, consistent with the previous studies (*SI Appendix*, Fig. S7). Thus, we conclude that in near-physiological (184 mM) ionic strength BRB80, positive charge in loop-12 contributes to the KIF1A run length, but in low (34 mM)-ionic strength BRB12, the loop swap has a negligible effect on the run length.

In contrast to the different run lengths, we found that KIF1A and KIF1A-SW in BRB80 had similar single-molecule microtubule landing rates of 0.055 ± 0.011 s^−1^·μM^−1^·μm^−1^ and 0.087 ± 0.026 s^−1^·μM^−1^·μm^−1^, respectively (mean ± 95% CI; Fig. 4*E*). Because this single-molecule landing rate method is highly sensitive to differences in relative activity between different motor preps, we performed complementary stopped-flow experiments to determine the apparent second-order rate constant for microtubule binding in BRB80. In this assay, KIF1A motors preincubated with mantADP are rapidly mixed with a range of microtubule concentrations in the presence of 1 mM ATP. Microtubule binding by KIF1A in the presence of excess ATP triggers mantADP release and a resulting decrease in mant fluorescence (16). Consistent with the single-molecule landing rates, the bimolecular on-rates of KIF1A (10.6 ± 0.5 μM^−1^s^−1^) and KIF1A-SW (9.6 ± 1.5 μM^−1^s^−1^) were similar (Fig. 4*F*).

To further investigate the interaction of the KIF1A loop-12 with microtubules, we removed the negatively charged C-terminal tails of tubulin by subtilisin proteolysis (*Materials and Methods* and *SI Appendix*, Fig. S8). We found that the average speed of KIF1A on subtilisin microtubules was unaffected (1.3 ± 0.39 μm/s; *SI Appendix*, Table S3), but the average run length was decreased by ~fivefold to 1.3 μm (*SI Appendix*, Fig. S9 and Table S3). Thus, decreasing the charge of the KIF1A loop-12 or cleaving the C-terminal tubulin tails had similar effects, implicating electrostatic interactions between these regions as an important contributor to the superprocessivity of KIF1A under zero load. In summary, at the near physiological (184 mM) ionic strength used in this study, the highly charged loop-12 is necessary for the unloaded superprocessivity of KIF1A, but it is not required for the initial strong-binding of KIF1A to microtubules.

### Both Loop-12 and the Nucleotide State of the Microtubule Affect the Load-Dependent Properties of KIF1A.

**Fig. 5.**
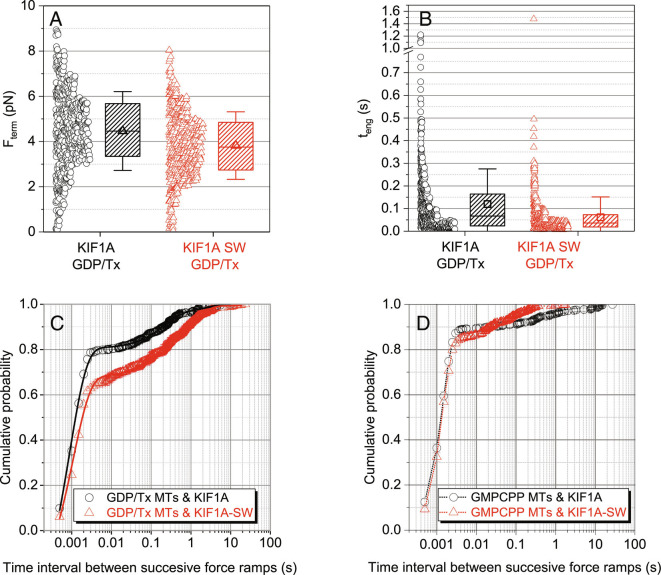
Influence of loop-12 and microtubule lattice on KIF1A performance under load. (*A*) Comparison of KIF1A and KIF1A-SW termination forces, F_term_ for primary events on taxol-stabilized microtubules. (*B*) Comparison of KIF1A and KIF1A-SW engagement times, t_teng_ for primary events on taxol-stabilized microtubules. (*C* and *D*): Cumulative probability distribution of time intervals between successive force ramps, t_restart_ for KIF1A (open circles) and KIF1A-SW (open triangles) on (*C*) taxol-stabilized and (*D*) GMPCCP-stabilized microtubules. Data include both primary and secondary events. Error bars are calculated using the bootstrap method (30), the solid lines in (*C*) represent fitting to three-exponential decay function (*Materials and Methods* and Table 1) and the dotted lines in (*D*) are just linear connections between the data points to serve as guide to the eye.

Given the importance of loop-12 for the unloaded processivity of KIF1A, we investigated the motile properties of KIF1A-SW under load. In the three-bead assay the median motor engagement duration, median-t_eng_, decreased from 0.069 s for KIF1A to 0.039 s for KIF1A-SW (Mann–Whitney test *P* < 0.001). Consistent with these shorter engagement times, the mean termination force, <F_term_>, decreased from 4.1 pN for KIF1A to 3.5 pN for KIF1A-SW (Fig. 5 and *SI Appendix*, Table S2). To determine if these lower termination forces were caused by differences in the motor stepping rate under load, force–velocity profiles were compared for KIF1A and KIF1A-SW and found to be similar (*SI Appendix*, Fig. S10). This similarity indicates that the lower mean termination force is a consequence of the shorter engagement duration. Taken together, when loop-12 was substituted, KIF1A exited the strong-binding state more readily under load. Interestingly, when subtilisin-treated microtubules were used in the three-bead assay to determine whether removal of the highly negatively charged C-terminal tails of tubulin (E-hook) produced a similar effect, we found that there was a large variability in the attachment duration for different microtubule dumbbells (*SI Appendix*, Fig. S11). Although it is unclear whether this variability is due to absence of the E-hooks, nonspecific cleavage of other regions of tubulin, or some other effect, we would like to draw caution to the use of subtilisin-treated microtubules, especially in loaded assays. Comparison with recombinant tubulin lacking C-terminal tails should elucidate this aspect in the future.

To investigate whether loop-12 of KIF1A contributes to the motor’s ability to rapidly reengage following termination of a force ramp, we quantified the time before reengagement, t_restart_ for the loop swap mutant. We found that 79% of KIF1A events reengaged within 2 ms, compared with only 66% of KIF1A-SW events (Fig. 5*C*). Coupled with the lack of effect on the unloaded landing rate (Fig. 4 *E* and *F*), positive charge in loop-12 does not significantly contribute to the initial interaction of the motor with the microtubule from solution, and it plays only a minor role in the fast reengagement with the microtubule following termination of a force ramp (Fig. 5*C*).

Our final investigation into the mechanism of fast reengagement kinetics of KIF1A asked whether properties of the microtubule lattice affect the KIF1A reengagement kinetics. Thus, instead of using taxol-stabilized GDP microtubules in the three-bead assay, we used microtubules polymerized with GMPCPP, which have differences in their microtubule lattice (31–34). The distributions of termination forces and engagement times were not substantially impacted for either KIF1A or KIF1A-SW on GMPCPP microtubules (Fig. 5 *A* and *B* and *SI Appendix*, Table S2), suggesting that the dissociation rate of KIF1A under load is not affected by the nucleotide state of the microtubule. However, the probability of restarting within 2 ms increased on GMPCPP microtubules relative to Taxol/GDP microtubules (Fig. 5 *C* and *D*). Strikingly, differences between the KIF1A and KIF1A-SW restart times that were observed on Taxol/GDP microtubules were abolished on GMPCPP microtubules. Shorter restart times on GMPCPP microtubules were also observed for KIF5B (*SI Appendix*, Fig. S12). Thus, the rate of reengagement with the microtubule under load is affected by i) the identity of the motor, ii) the presence of the loop-12, and iii) the nucleotide state of the microtubule lattice.

## Discussion

The ability of kinesin motors to power intracellular transport against mechanical loads is integral to their function. The influence of load on motor speed and microtubule attachment lifetimes has been characterized using optical tweezers for a number of kinesin isoforms (e.g., refs. 10 and 35–37). However, little is known about the load-dependence of kinesin-3 motility, which is of particular interest given its superprocessive behavior under zero load. Here, we find that KIF1A processive runs are readily terminated under load, resulting in lower average termination forces as compared with KIF5B. However, this behavior is compensated for by a rapid reengagement of the motor and recovery of its force-generating capacity, which is particularly apparent in the three-bead assay. These rapid KIF1A reengagement kinetics, also observed in a recent single-bead trap studies (15, 38), are consistent with the fast bimolecular association rate constant for microtubule binding reported in a recent biochemical study (16). KIF1A therefore represents a different paradigm than KIF5B for an efficient transporter under force by rapidly and repeatedly reengaging with the microtubule and restarting its processive motion. Thus, whereas KIF1A is superprocessive in the absence of load, under load it may be better characterized as superengaging.

### Performance of KIF1A under Load.

By implementing the three-bead assay in a dual-beam optical tweezer setup, we were able to investigate the performance of KIF1A as it stepped against loads oriented primarily parallel to the microtubule long axis. Importantly, we found that that KIF1A forces, although somewhat smaller on average, are comparable to those generated by KIF5B. KIF1A did not generate long-lived (>0.2 s) force plateaus, or “stalls” seen frequently with KIF5B in the three-bead assay (20); instead, KIF1A force ramps terminated before reaching a plateau more frequently. Thus, instead of quantifying a “stall force”, we quantified the force at the termination of force ramps, F_term_, and found that in both the single- and three-bead assays, <F_term_> was smaller for KIF1A than for KIF5B. The lower KIF1A termination forces reflect the inability of KIF1A to remain strongly engaged with the microtubule under load, which may be a useful adaptation to achieve bidirectional motion (discussed below). Interestingly, the engagement times and termination forces for both KIF1A and KIF5B are smaller in the single-bead rather than in the three-bead assay, which demonstrates that vertical loads accelerate termination of force ramps for these kinesin isoforms.

### Mechanism of Fast KIF1A Reengagement.

A distinct feature of KIF1A motor behavior is its fast reengagement with the microtubule following the termination of a force ramp. Almost 79% of reengagements for KIF1A in the three-bead assay occurred within 2 ms, compared with only 20% for KIF5B (Fig. 3*B*). Consensus models for the kinesin chemomechanical cycle point to the motor being in a weak-binding ADP-P_i_ or adenosine diphosphate (ADP) state at the termination of the force ramp, and the transition to the strong-binding state to start the next force ramp requiring ADP release to generate the tight-binding apo state (39). Furthermore, two recent kinesin-1 optical trapping studies characterized fast unbinding and rebinding events that occur while kinesin-1 slides backward after force ramp termination (26, 27). Toleikis et al. found that during stall plateaus the bead slipped backward in 8 nm and longer displacements (26). Dwell times preceding backward displacements were longer than those preceding forward steps, consistent with the motor releasing P_i_ and slipping backward in the ADP state. Using a small, high refractive index bead, Sudhakar et al. found that during the backslipping process, the bead paused transiently (~30 μs) at 8-nm increments, consistent with the motor interacting transiently with successive tubulin subunits as it slid backward along the protofilament (27). Both studies concluded that under load, kinesin-1 can enter a weakly bound ADP or ADP-P_i_ state and slip backward along the microtubule, and then reengage and recover. The larger drag coefficient of the microtubule dumbbell in our three-bead assay masks detection of microsecond interactions between KIF1A and the microtubule during the backward displacements. However, the millisecond-scale rescue of processive motion that we observe is consistent with KIF1A entering a weak-binding or “slip” state like KIF5B, but transitioning back to a strong-binding, force-generating state much faster than KIF5B.

**Fig. 6.**
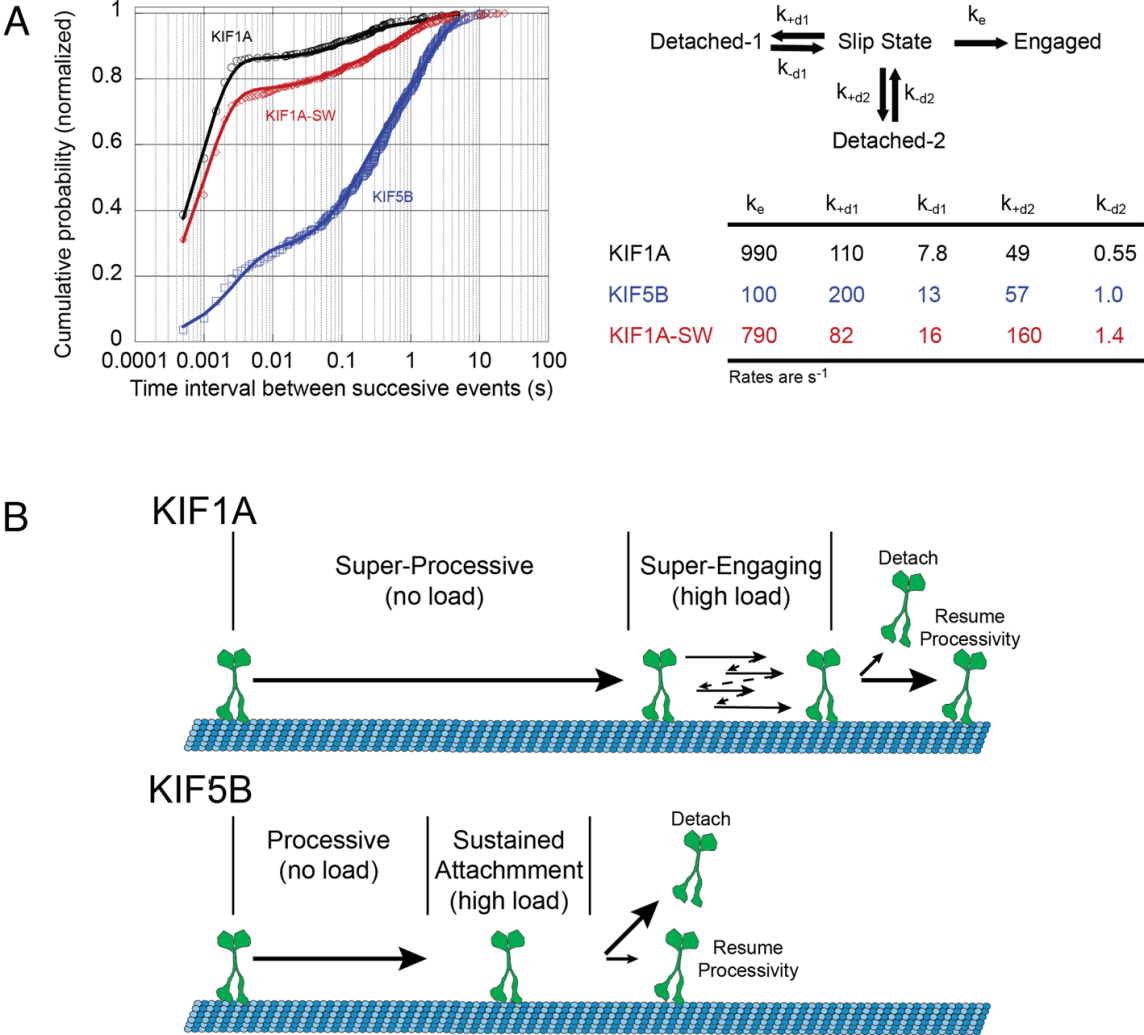
Comparison of reengagement rates between KIF1A and KIF5B. (*A*): Cumulative probability distribution of time intervals between successive force ramps, t_restart_ for KIF1A (black open circles), KIF1A-SW (red open triangles), and KIF5B (blue open squares) on taxol-stabilized microtubules. Data are offset to account for missed events resulting from the 0.5-ms minimum detection limit. Solid lines are fits to model of reengagement kinetics. Kinetic model of motor reengagement. Following termination of a force ramp, the motor is in a Slip state. The motor can then reengage with the microtubule, with rate k_e_, or it can detach from the microtubule with two different rates k_+d1_ and k_+d2_ that depend on the motor–microtubule geometry and other factors. From the detached state, the motor can return to the slip state with rates k_-d1_ and k_-d2_ and reengage with the microtubule. Table shows the fits of the kinetic model to the t_restart_ times for the three motors. (*B*) A schematic description showing the higher propensity of KIF1A relative to KIF5B to reengage with the microtubule track instead of detaching after experiencing opposing loads.

To explore the kinetics of this reengagement process, we constructed a kinetic model and fit it to our normalized cumulative distributions of restart times for KIF1A, KIF1A-SW, and KIF5B. Following termination of a force ramp, the motor starts in a weakly bound Slip state and can then either transition to an Engaged state and continue to step against the load, or it can dissociate and enter a Detached state (Fig. 6). Our experimental t_restart_ times (Figs. 3 and 5) correspond to the time it takes to transition from the Slip state to the Engaged state. To account for the two slower time constants in the t_restart_ distributions, we included two *Detached* states; we hypothesize that transitions into and out of these detached states are influenced by the bead geometry and other experimental uncertainties (see below). Note that in the model, the rate of the fast reengagement population as well as the relative proportion of fast reengagement events is determined by a kinetic race between the engagement rate constant, k_e_, and the two detachment rate constants, k_+d1_ and k_+d2_.

When we fit the model to the experimental data, the KIF5B reengagement rate k_e_ was 100 s^−1^, whereas the KIF1A reengagement rate was 990 s^−1^. Transition into the strongly bound state is thought to be limited by ADP release (39). Published values for the ADP release rate of KIF5B in the absence of external loads range from 110 to 306 s^−1^ (40–43), which is close to the estimated k_e_ from our model (Fig. 6). However, the estimated value of k_e_ for KIF1A is more than twice the reported rate of ~350 s^−1^ for the ADP release when KIF1A is bound to the microtubule in a one-headed state in the absence of external load (16). How can we account for this fast KIF1A reengagement rate? One possibility is that this transition is load dependent, such that rearward load on the motor when it engages with the microtubule accelerates ADP release and thus the transition to a strong-binding state. Another consideration is that in the three-bead assay, the microtubule is under tensile forces even in the absence of interactions with kinesin; these tensile forces could alter the microtubule lattice in a way that enhances KIF1A engagement kinetics and/or ADP release. A third possibility is that KIF1A detaches in a nucleotide-free strong-binding state and is able to rapidly reengage without needing to release ADP or undergo the subsequent weak-to-strong transition. Additional experiments will be required to distinguish among these possibilities.

### The Role of the Loop-12 in KIF1A Motility.

A distinctive feature of the KIF1A sequence is the positively charged K-loop insert of loop-12, but the role of this region in KIF1A motility under load remains murky. The importance of electrostatic interactions mediated by this loop was established in work on recombinant KIF1A monomers where it was shown that diffusive tethering by the K-loop enabled processivity (5, 6). In later work, KIF1A dimers were shown to be superprocessive in low (34 mM)-ionic strength BRB12 buffer (7, 8). As part of this work, it was found that replacing the lysines in loop-12 of KIF1A with the analogous sequence from kinesin-1 (KIF5C) did not abolish the superprocessivity at low-ionic strength, but it decreased the microtubule landing rate in the absence of force. We find here that in BRB80, substituting the KIF1A loop-12 with that of *Drosophila* kinesin-1 decreases the unloaded run length sixfold, and deleting the C-terminal tails of tubulin has a similar effect. We propose that the previously observed insensitivity of run length to swapping Loop-12 (8) resulted from the reversible dimerization of these constructs, which lacked a stabilizing leucine zipper domain (7, 8). This reversal of dimerization may have prematurely terminated run lengths, masking any effects of swapping loop-12 (11). Importantly, we found that swapping loop-12 in BRB80 only marginally affected the initial microtubule landing rate in the absence of load, as assessed by single-molecule TIRF (Fig. 4*E*) and ensemble stopped-flow measurements (Fig. 4*F*). Thus, when a motor in solution first encounters a microtubule, transition to the strong-binding state and ADP release is mediated by interaction of the canonical microtubule binding site (44) with the microtubule, rather than through initial formation of a tethered intermediate that is stabilized by the K-loop.

In the presence of hindering loads, we found that swapping the kinesin-1 loop-12 into KIF1A had only minor effects on the ability of KIF1A to remain engaged with the microtubule. For instance, in the three-bead assay, swapping loop-12 reduced <F_term_> by ~25% and median-t_eng_ by ~twofold. Similarly, reengagement kinetics were minimally affected; in the three-bead assay, the fraction of reengagement events that occurred within 2 ms decreased by only ~25% for KIF1A-SW, which is broadly consistent with the lack of an effect of the K-loop on the landing rate from solution. These results have important implications for understanding the unique mechanism by which KIF1A sustains motility in the presence of obstacles and resisting mechanical loads.

A recent KIF1A single-bead optical tweezer study using the same protein construct found that removal of the K-loop led to a nearly sevenfold decrease in the number of force events per microtubule encounter (13). Although our results appear to conflict with this work, there are a number of differences between the two studies that can explain the discrepancy. Our findings agree with the finding of Budaitis et al that KIF1A can rapidly reattach to the microtubule after detaching under load in the single-bead assay (Fig. 3*B*), but we expanded this result to show that rapid (<2 ms) reengagements are even more likely in the three-bead assays (Fig. 3*A*). Importantly, the three-bead assay geometry better controls the proximity of KIF1A to the microtubule because the motor is immobilized on a stable pedestal, whereas in the single-bead assay, the bead is free to rotate after the motor detaches. One possibility is that in the single-bead assay, positive charge in the K-loop keeps the motor near the microtubule, preventing bead rotation and allowing faster motor reengagement, and removing this charge eliminates this stabilizing influence. A second issue is that the number of force events per microtubule encounter depends both on the rate of reengagement as well as the force ramp termination kinetics. It is possible that vertical forces inherent in the single-bead geometry affect the rates of the various detachment processes and thus affect quantification of force events per microtubule encounter. Finally, in both studies there was qualitative agreement that the behavior of KIF1A-SW was intermediate between KIF1A and the kinesin-1 control.

Intriguingly, when we quantified reengagement kinetics on microtubules polymerized in GMPCPP, which have been shown to have different lattices compared with Taxol/GDP microtubules (31, 33, 34), the proportion of rapid reengagement events increased for KIF1A-SW and matched that of wild type. A recent study examining delivery of vesicles to synaptic boutons found that KIF1A has a lower affinity in vitro for GMPCPP microtubules compared with GDP/taxol microtubules (45). That reduced affinity was not observed in our measurements, but there are a number of differences between the assays, most notably load and concentrations of motors (single molecule versus saturating).

Overall, we find that the positive charge in loop-12 strongly contributes to the unloaded superprocessivity of KIF1A, but it plays only a minor role in the termination and reengagement kinetics under load.

### Physiological Relevance of the Optical Trapping Geometries.

According to recent experimental (20) and theoretical (19) studies, the vertical force components inherent to the single-bead assay accelerated termination of the KIF5B force ramps leading to an underestimation of t_eng_ and F_term_. Additionally, application of the three-bead assay revealed a 10-fold microtubule-to-microtubule variability in t_eng_. Thus, it was important to evaluate KIF1A motility with both the single- and three-bead optical trapping assays. Like KIF5B, we observed larger t_eng_ and F_term_ values for KIF1A using the three-bead geometry, but the microtubule–to-microtubule variability was not seen, indicating that this is a special feature of KIF5B.

In the cellular environment, kinesin can experience hindering loads at various angles depending on the relative orientation between the microtubule track and the vector of the resisting force applied on the cargo. However, during axonal and dendritic transport, the majority of the microtubule tracks are parallel to each other, and motility is largely restricted to one dimension. The vector of any hindering force opposing transport is thus expected to be predominantly parallel to the microtubule for two reasons. First, the measured mechanical properties of intracellular organelles, including axonal cargoes (46, 47), indicate that they are deformable in response to motor-generated forces. This is consistent with in vivo observations of motor-driven tubulation of organelles such as mitochondria and autolysosomes (48, 49). Second, for cargoes with radii smaller than the length of kinesin motors [~60 nm (50)], such as synaptic vesicles and small dense core vesicles [20 to 30 nm radii (51–53)] the vertical force component F_z_ is roughly one third of F_x_.

Although the use of the single-bead assay results in the underestimation of t_eng_ and F_term_, its comparison with the three-bead assay provides unique insights into the effect of vertical forces on kinesin motility (20). Notably in a previous study (15), removing charges in loop-12 of KIF1A substantially affected the number of force events per microtubule encounter measured in the single-bead assay, yet in the three-bead assay in the current study, this results in only minor effects. It is only by comparison of these assays did we learn that loop-12 charges may prevent the rotation of the cargo away from the microtubule after termination of a force ramp, facilitating rapid motor association.

### Insights into the Biological Function of KIF1A.

The principal role of KIF1A in cells is vesicle transport and, unlike KIF5A, which transports cargo exclusively in axons, KIF1A transports cargo in both axons and dendrites (54–56). Much of this transport is bidirectional (57), meaning that KIF1A must both navigate diverse microtubule substrates, but also transport cargo against hindering loads generated by dynein. Although KIF1A has been characterized as a superprocessive motor in the absence of load, it is clear from Budaitis et al. (15) and our work that mechanical load more easily ends these processive runs, compared with KIF5B.

KIF1A has evolved kinetic features that allow it to be superengaging. First, KIF1A has a 10-fold faster bimolecular on-rate from solution, compared with kinesin-1 (16, 58). This fast initial binding rate is mediated at near physiological ionic strength not by the highly charged K-loop, but rather by other structural and mechanochemical features of the catalytic domain (17). Second, following force ramp termination, KIF1A is able to rapidly reengage with the microtubule and resume forward stepping. These features confer a distinct advantage during intracellular transport because they increase the probability that when a motor detaches from the microtubule it will rapidly reengage to continue transport. A recent study that tethered kinesin-1 and kinesin-3 motors to dynein-dynactin-BicD2 complexes found that KIF1A could compete against dynein nearly as effectively as kinesin-1, despite their different load-dependent off-rates (59). Importantly, a computational model that incorporated slow (5 s^−1^) (58, 60) reattachment rates for KIF1A and kinesin-1 was unable to recapitulate the data, but if the reengagement rates were set to 990 s^−1^ for KIF1A and to 100 s^−1^ for kinesin-1 from Fig. 6, the model was able to reproduce the experimental data. Thus, despite the propensity of KIF1A to readily detach under load, its fast reengagement kinetics allows it to mechanically compete with dynein during bidirectional transport. Furthermore, compared with the slower detachment/reattachment kinetics of kinesin-1, the rapid reversible binding strategy may enable KIF1A to better navigate around roadblocks such as MAPs and other obstacles on the microtubule.

## Materials and Methods

### Protein Constructs and Purification.

The KIF1A-WT construct [adapted from Addgene #61665 (12)] consists of the *R. norvegicus* KIF1A residues 1 to 393, followed by a GCN4 leucine zipper for dimerization and an eGFP tag. The KIF1A-SW was modified by swapping the native loop-12 (residues 288 to 308) of the KIF1A construct with the *Drosophila melanogaster* KHC loop-12 sequence (GNKTHIPYRD). This *D. melanogaster* loop-12 sequence was used because it provides a direct comparison to previous work (11, 16), and it changes the charge of the loop with less sequence divergence than using loop-12 from KIF5B. Both constructs have a C-terminal His tag and were bacterially expressed and purified by nickel gravity column chromatography, as described previously (16). The elution buffer, consisting of 20 mM phosphate buffer, 500 mM sodium chloride, 500 mM imidazole,10 μM ATP, and 5 mM DTT was supplemented with 10% glycerol before flash freezing and storing at −80 °C. Concentrations were determined using GFP absorbance at 488 nm.

### Chemical Reagents and Microspheres.

Unlabeled porcine tubulin and its labeled analogs, (TRITC and biotin), GTP and Paclitaxel were purchased from Cytoskeleton, Inc. Mouse monoclonal anti-6xHis tag antibody and rat tubulin antibody which recognizes the C-terminal tail of α tubulin were purchased from ABCAM. GMPCPP was purchased from Jena Biosciences, Germany. Streptavidin-coated polystyrene beads 1% w/v (0.82 μm in diameter) and silica microspheres 9,92 % solid w/v (5.0 μm in diameter) were purchased from Spherotech. Amyl acetate and 2% Colloidon in amyl acetate were purchased from Electron Microscopy Sciences. Glass coverslips 22 × 45 × 1.5 mM were purchased from Fisher Scientific. Glucose oxidase from *Aspergillous niger*, aqueous solution of catalase from bovine liver, dimethyl sulfoxide (DMSO), phenylmethylsulfonyl fluoride (PMSF), ATP, MgCl_2,_ and Subtilisin A *Bacillus licheniformis* were purchased from Sigma Aldrich. Mouse anti-tubulin b3 antibody which recognizes the C-terminal tail of β tubulin was purchased from Bio-Rad Laboratories. Except where noted, all experiments were carried out in BRB80 buffer (80 mM PIPES, 1 mM MgCl_2_, 1mM EGTA, 2 mM MgATP, pH 6.9, 184 mM ionic strength). A subset of single-molecule TIRF measurements were carried out in BRB12 buffer (12 mM PIPES, 1 mM MgCl_2_, 1mM EGTA, 2 mM MgATP, pH 6.9, 36 mM ionic strength). Detailed ionic strength calculations are included in *SI Appendix*.

### Optical Tweezer Experiments.

Taxol-stabilized GDP microtubules and GMPCPP-stabilized microtubules were prepared from nonpolymerized porcine tubulin as previously described (20). For the single-bead assay, 4% TRITC-tubulin was included, while for the three-bead assay 4% Tetramethylrhodamine (TRITC) tubulin as well as 48% biotinylated tubulin were included.

For the single-bead assay, nitrocellulose-coated coverslips were assembled into flow chambers of 20 μL volume as described previously (20), and used within 24 h of preparation. Aqueous solutions in BRB80 were introduced in the chamber in the following sequence: 20 μL of 0.05 mg/mL anti-tubulin antibody (Bio-Rad Laboratories) for 5 min, 50 μL of 2 mg/mL casein for 4 min, 4 × 25 μL of 125 nM 4% TRITC microtubules supplemented with 2 mg/mL casein and 20 mM taxol for 4 × 1 min, wash with 100 μL of 2 mg/mL casein, and 50 μL of final solution containing kinesin beads, 2 mM ATP, 2 mM MgCl_2_, 50 mM DTT, 20 μM taxol, 5 mg/mL glucose, 1,500 units/mL glucose oxidase, and 0.2 units/mL catalase. The open ends of the flow chamber were sealed with vacuum grease to prevent evaporation during the experiment. To ensure single-molecule interactions, concentrations of kinesin were used such that no more than one out of three kinesin-decorated beads interacted with surface immobilized microtubules.

For the three-bead assay, a solution of silica spherical pedestals (dia. 5.0 μm) was dried on a coverslip, coated with nitrocellulose-film, and assembled into ~ 20-μL flow chambers, as previously described (20). Aqueous solutions in BRB80 were introduced into the flow chamber in the following sequence: 20 μL of 0.2 mg/mL anti-6xHis antibody (Abcam) for 5 min, 50 μL of 2 mg/mL casein for 4 min, 50 μL of kinesin construct ~1 nM supplemented with 2 mg/mL casein for 5 min, 100 μL of 2 mg/mL casein wash, and 50 μL of final solution containing 5 nM 48% biotinylated-4% TRITC microtubules, 2 mM ATP, 2 mM MgCl_2_, 50 mM DTT, 20 μM taxol (excluded when GMPCPP microtubules were used), 5 mg/mL glucose, 1,500 units/mL glucose oxidase, and 0.2 units/mL catalase. Before sealing the chamber with vacuum grease, 3 to 4 μL streptavidin beads (dia. 0.82 µm) diluted 1:30 in the final solution without microtubules were introduced from one side of the chamber. To ensure single-molecule interactions, concentrations of kinesin were used such that no more than one out of three kinesin-decorated spherical immobilized pedestals interacted with microtubule dumbbells.

### Optical Tweezer Instrumentation and Data Analysis.

We used a custom-made dual-laser beam (1,064 nm) optical trap system equipped with a 63× water objective, 1.2 numerical aperture as previously described (20). The trap stiffness (pN/nm) and the system-calibration factor (pN/V) for each trapped bead were determined in the absence of any microtubule interaction by calculating and fitting to a Lorentzian function the power spectrum of the Brownian motion of the beads in the trap. Microtubule dumbbells were subjected to stretching forces of 4 to 5 pN by moving the two laser beams apart. The trap stiffness of the individual laser beams for single-bead assays was 0.04 to 0.12 pN/nm and for three-bead assays was 0.060 to 0.090 pN/nm. The higher total stiffness in the three-bead assay was required to accommodate the sum of the stretching forces on the microtubule dumbbell and the forces generated by kinesin. The higher stiffness also decreases the relaxation time of the dumbbell close to the relaxation time of the bead in the single-bead assay (*SI Appendix*). Since the laser traps are stationary, a piezoelectric stage controller was used to move the flow chamber and therefore control the relative position between single beads and surface-immobilized microtubules or between microtubule dumbbells and surface-immobilized spherical pedestals. Data were digitized at a scanning rate of 2 kHz and filtered at 1 kHz using in-house software written in LabVIEW. A termination event was defined when a strictly monotonic decrease in force was larger than the SD of 3-ms windows immediately before or after the event.

For data analysis, in-house software written in LabVIEW was used, while for statistical analysis, curve fitting and graphs Origin 2018b software was used, as described previously (20). The cumulative probability distributions for the time intervals between successive force ramps were fit using the triexponential decay function:

$$P\left(t\right)=A_{0}-\overset{3}{\underset{i=1}{\sum }}A_{i}e^{\frac{\left(t-t_{0}\right)}{\tau_{i}}}.$$

All the parameters were free, except *t*_0_ which was set equal to 0.5 ms and corresponds the temporal resolution of the optical tweezer data. The final amplitude values reported in Table 1 are relative values divided by their total sum ΣA_i_ such that the probability density is normalized to one over the observed range of values t ≥ t_0_ instead of t ≥ 0, and A_0_ = 1 (30, 61, 62). The kinetic modeling in Fig. 6 was done using the kinetics simulator Tenua (http://bililite.com/tenua/).

### TIRF Experiments.

Single-molecule tracking experiments of GFP-labeled KIF1A-WT and KIF1A-SW were performed on a Nikon TE2000 TIRF microscope at 21 °C, as described previously (43, 63, 64). Flow cells were prepared by flowing in 2 mg/mL casein, followed by full-length rigor kinesin (43) and taxol-stabilized, Cy5 (GE Healthcare)-labeled microtubules. The microtubules were incubated for 30 s, followed by a wash, and repeated 2×. Motors were diluted to 200 to 500 pM and added to the flow cell in the presence of 2 mM ATP and imaged at 5 fps. The kymographs were analyzed manually using Fiji (NIH) (65) to determine the run lengths, velocities, and landing rates. Motor intensities were determined by taking the mean intensity for each trace from the kymographs and subtracting the background.

### Stopped Flow Experiments.

Stopped-flow experiments were performed using an Applied Photophysics SX20 spectrofluorometer at 25 °C in BRB80 buffer, as previously described (16, 66). For k_on_^Mt^ measurements, a solution of 150 nM motor dimers and 0.25 mM free mADP was flushed against a solution containing 5 μM Taxol, 1 mM ATP, varying concentrations of taxol-stabilized microtubules (all final chamber concentrations). After mixing, mADP released from the bound head produced a decrease in fluorescence at 356 nm, which was fit with a single exponential to determine the k_obs_ at each microtubule concentration. The averaged trace of five to seven consecutive shots was fit and reported for each trial. Linear fit to the rates versus the microtubule concentration gives the bimolecular on-rate (16, 66).

## Supplementary Material

Appendix 01 (PDF)

## Data Availability

All study data included in the article and/or *SI Appendix* are available online at zenodo.org, DOI: 10.5281/zenodo.7448351.

## References

[1] Y. Okada, H. Yamazaki, Y. Sekine-Aizawa, N. Hirokawa, The neuron-specific kinesin superfamily protein KIF1A is a unique monomeric motor for anterograde axonal transport of synaptic vesicle precursors. Cell **81**, 769–780 (1995). 7539720 10.1016/0092-8674(95)90538-3

[2] N. Hirokawa, Y. Noda, Y. Tanaka, S. Niwa, Kinesin superfamily motor proteins and intracellular transport. Nat. Rev. Mol. Cell Biol. **10**, 682–696 (2009). 19773780 10.1038/nrm2774

[3] K. Chiba , Disease-associated mutations hyperactivate KIF1A motility and anterograde axonal transport of synaptic vesicle precursors. Proc. Natl. Acad. Sci. U.S.A. **116**, 18429–18434 (2019). 31455732 10.1073/pnas.1905690116 PMC6744892

[4] M. Pennings , KIF1A variants are a frequent cause of autosomal dominant hereditary spastic paraplegia. Eur. J. Hum. Genet. **28**, 40–49 (2020). 31488895 10.1038/s41431-019-0497-z PMC6906463

[5] Y. Okada, N. Hirokawa, A processive single-headed motor: Kinesin superfamily protein KIF1A. Science **283**, 1152–1157 (1999). 10024239 10.1126/science.283.5405.1152

[6] Y. Okada, N. Hirokawa, Mechanism of the single-headed processivity: Diffusional anchoring between the K-loop of kinesin and the C terminus of tubulin. Proc. Natl. Acad. Sci. U.S.A. **97**, 640–645 (2000). 10639132 10.1073/pnas.97.2.640 PMC15383

[7] V. Soppina , Dimerization of mammalian kinesin-3 motors results in superprocessive motion. Proc. Natl. Acad. Sci. U.S.A. **111**, 5562–5567 (2014). 24706892 10.1073/pnas.1400759111 PMC3992690

[8] V. Soppina, K. J. Verhey, The family-specific K-loop influences the microtubule on-rate but not the superprocessivity of kinesin-3 motors. Mol. Biol. Cell **25**, 2161–2170 (2014). 24850887 10.1091/mbc.E14-01-0696 PMC4091829

[9] D. V. Lessard , Polyglutamylation of tubulin’s C-terminal tail controls pausing and motility of kinesin-3 family member KIF1A. J. Biol. Chem. **294**, 6353–6363 (2019). 30770469 10.1074/jbc.RA118.005765 PMC6484136

[10] M. Tomishige, D. R. Klopfenstein, R. D. Vale, Conversion of Unc104/KIF1A kinesin into a processive motor after dimerization. Science **297**, 2263–2267 (2002). 12351789 10.1126/science.1073386

[11] T. M. Zaniewski, W. O. Hancock, The net charge of the K-loop regulates KIF1A superprocessivity by enhancing microtubule affinity in the one-head-bound state. bioRxiv [Preprint] (2022). 10.1101/2022.08.21.504701. Accessed 21 August 2022. PMC9871336 36549649

[12] S. R. Norris , A method for multiprotein assembly in cells reveals independent action of kinesins in complex. J. Cell Biol. **207**, 393–406 (2014). 25365993 10.1083/jcb.201407086 PMC4226728

[13] G. Arpag, S. Shastry, W. O. Hancock, E. Tuzel, Transport by populations of fast and slow kinesins uncovers novel family-dependent motor characteristics important for in vivo function. Biophys. J. **107**, 1896–1904 (2014). 25418170 10.1016/j.bpj.2014.09.009 PMC4213720

[14] G. Arpag , Motor dynamics underlying cargo transport by pairs of kinesin-1 and kinesin-3 motors. Biophys. J. **116**, 1115–1126 (2019). 30824116 10.1016/j.bpj.2019.01.036 PMC6428962

[15] B. G. Budaitis , Pathogenic mutations in the kinesin-3 motor KIF1A diminish force generation and movement through allosteric mechanisms. J. Cell Biol. **220**, e202004227 (2021). 33496723 10.1083/jcb.202004227 PMC7844421

[16] T. M. Zaniewski, A. M. Gicking, J. Fricks, W. O. Hancock, A kinetic dissection of the fast and superprocessive kinesin-3 KIF1A reveals a predominant one-head-bound state during its chemomechanical cycle. J. Biol. Chem. **295**, 17889–17903 (2020). 33082143 10.1074/jbc.RA120.014961 PMC7939386

[17] G. Scarabelli , Mapping the processivity determinants of the kinesin-3 motor domain. Biophys. J. **109**, 1537–1540 (2015). 26488644 10.1016/j.bpj.2015.08.027 PMC4624112

[18] J. Ren , Structural delineation of the neck linker of kinesin-3 for processive movement. J. Mol. Biol. **430**, 2030–2041 (2018). 29752968 10.1016/j.jmb.2018.05.010

[19] H. Khataee, J. Howard, Force generated by two kinesin motors depends on the load direction and intermolecular coupling. Phys. Rev. Lett. **122**, 188101–188106 (2019). 31144901 10.1103/PhysRevLett.122.188101 PMC12175961

[20] S. Pyrpassopoulos, H. Shuman, E. M. Ostap, Modulation of kinesin’s load-bearing capacity by force geometry and the microtubule track. Biophys. J. **118**, 243–253 (2020). 31883614 10.1016/j.bpj.2019.10.045 PMC6952184

[21] M. J. deCastro, R. M. Fondecave, L. A. Clarke, C. F. Schmidt, R. J. Stewart, Working strokes by single molecules of the kinesin-related microtubule motor ncd. Nat. Cell Biol. **2**, 724–729 (2000). 11025663 10.1038/35036357

[22] J. W. Hammond , Mammalian kinesin-3 motors are dimeric in vivo and move by processive motility upon release of autoinhibition. PLoS Biol. **7**, e72 (2009). 19338388 10.1371/journal.pbio.1000072 PMC2661964

[23] S. Niwa , Autoinhibition of a neuronal kinesin UNC-104/KIF1A regulates the size and density of synapses. Cell Rep. **16**, 2129–2141 (2016). 27524618 10.1016/j.celrep.2016.07.043 PMC5432123

[24] K. Svoboda, C. F. Schmidt, B. J. Schnapp, S. M. Block, Direct observation of kinesin stepping by optical trapping interferometry. Nature **365**, 721–727 (1993). 8413650 10.1038/365721a0

[25] A. Jannasch, V. Bormuth, M. Storch, J. Howard, E. Schaffer, Kinesin-8 is a low-force motor protein with a weakly bound slip state. Biophys. J. **104**, 2456–2464 (2013). 23746518 10.1016/j.bpj.2013.02.040 PMC3672894

[26] A. Toleikis, N. J. Carter, R. A. Cross, Backstepping mechanism of kinesin-1. Biophys. J. **119**, 1984–1994 (2020). 33091340 10.1016/j.bpj.2020.09.034 PMC7732724

[27] S. Sudhakar , Germanium nanospheres for ultraresolution picotensiometry of kinesin motors. Science **371**, eabd9944 (2021). 33574186 10.1126/science.abd9944

[28] S. Pyrpassopoulos, H. Shuman, E. M. Ostap, Microtubule dumbbells to assess the effect of force geometry on single kinesin motors. Methods Mol. Biol. **2478**, 559–583 (2022). 36063334 10.1007/978-1-0716-2229-2_20 PMC9987583

[29] K. Sekimoto, J. Prost, Elastic anisotropy scenario for cooperative binding of kinesin-coated beads on microtubules. J. Phys. Chem. B **120**, 5953–5959 (2016). 27027685 10.1021/acs.jpcb.6b01627

[30] W. H. Presse, P. B. Flannery, S. A. Teukolsky, W. T. Vetterling, Numerical Recipes in C: The Art of Scientific Computing (Cambridge University Press, Cambridge, U.K., ed. 2, 1988).

[31] G. M. Alushin , High-resolution microtubule structures reveal the structural transitions in alphabeta-tubulin upon GTP hydrolysis. Cell **157**, 1117–1129 (2014). 24855948 10.1016/j.cell.2014.03.053 PMC4054694

[32] R. Zhang, G. M. Alushin, A. Brown, E. Nogales, Mechanistic origin of microtubule dynamic instability and its modulation by EB proteins. Cell **162**, 849–859 (2015). 26234155 10.1016/j.cell.2015.07.012 PMC4537847

[33] R. Zhang, B. LaFrance, E. Nogales, Separating the effects of nucleotide and EB binding on microtubule structure. Proc. Natl. Acad. Sci. U.S.A. **115**, E6191–E6200 (2018). 29915050 10.1073/pnas.1802637115 PMC6142192

[34] J. Estevez-Gallego , Structural model for differential cap maturation at growing microtubule ends. Elife **9**, e50155 (2020). 32151315 10.7554/eLife.50155 PMC7064335

[35] K. Visscher, M. J. Schnitzer, S. M. Block, Single kinesin molecules studied with a molecular force clamp. Nature **400**, 184–189 (1999). 10408448 10.1038/22146

[36] M. T. Valentine, P. M. Fordyce, T. C. Krzysiak, S. P. Gilbert, S. M. Block, Individual dimers of the mitotic kinesin motor Eg5 step processively and support substantial loads in vitro. Nat. Cell Biol. **8**, 470–476 (2006). 16604065 10.1038/ncb1394 PMC1523314

[37] J. O. Andreasson, S. Shastry, W. O. Hancock, S. M. Block, The mechanochemical cycle of mammalian kinesin-2 KIF3A/B under Load. Curr. Biol. **25**, 1166–1175 (2015). 25866395 10.1016/j.cub.2015.03.013 PMC4422762

[38] A. J. Lam , A highly conserved 310 helix within the kinesin motor domain is critical for kinesin function and human health. Sci. Adv. **7**, eabf1002 (2021). 33931448 10.1126/sciadv.abf1002 PMC8087401

[39] W. O. Hancock, The kinesin-1 chemomechanical cycle: Stepping toward a consensus. Biophys. J. **110**, 1216–1225 (2016). 27028632 10.1016/j.bpj.2016.02.025 PMC4816755

[40] S. P. Gilbert, M. R. Webb, M. Brune, K. A. Johnson, Pathway of processive ATP hydrolysis by kinesin. Nature **373**, 671–676 (1995). 7854446 10.1038/373671a0 PMC1855160

[41] K. M. Brendza, C. A. Sontag, W. M. Saxton, S. P. Gilbert, A kinesin mutation that uncouples motor domains and desensitizes the gamma-phosphate sensor. J. Biol. Chem. **275**, 22187–22195 (2000). 10767290 10.1074/jbc.M001124200 PMC1560104

[42] D. D. Hackney, Pathway of ADP-stimulated ADP release and dissociation of tethered kinesin from microtubules. implications for the extent of processivity. Biochemistry **41**, 4437–4446 (2002). 11914091 10.1021/bi0159229

[43] K. J. Mickolajczyk , Kinetics of nucleotide-dependent structural transitions in the kinesin-1 hydrolysis cycle. Proc. Natl. Acad. Sci. U.S.A. **112**, E7186–7193 (2015). 26676576 10.1073/pnas.1517638112 PMC4702989

[44] G. Woehlke , Microtubule interaction site of the kinesin motor. Cell **90**, 207–216 (1997). 9244295 10.1016/s0092-8674(00)80329-3

[45] P. Guedes-Dias , Kinesin-3 responds to local microtubule dynamics to target synaptic cargo delivery to the presynapse. Curr. Biol. **29**, 268–282.e268 (2019). 30612907 10.1016/j.cub.2018.11.065 PMC6342647

[46] J. Saurabh, A versatile mitochondria isolation- and analysis-pipeline generates 3D nano-topographies and mechano-physical surface maps of single organelles. bioRxiv [Preprint] (2021). 10.1101/2021.10.31.466655. Accessed 31 October 2021.

[47] D. Mahecic , Mitochondrial membrane tension governs fission. Cell Rep. **35**, 108947 (2021). 33852852 10.1016/j.celrep.2021.108947

[48] C. Wang , Dynamic tubulation of mitochondria drives mitochondrial network formation. Cell Res. **25**, 1108–1120 (2015). 26206315 10.1038/cr.2015.89 PMC4650629

[49] W. Du , Kinesin 1 drives autolysosome tubulation. Dev. Cell **37**, 326–336 (2016). 27219061 10.1016/j.devcel.2016.04.014

[50] J. Kerssemakers, J. Howard, H. Hess, S. Diez, The distance that kinesin-1 holds its cargo from the microtubule surface measured by fluorescence interference contrast microscopy. Proc. Natl. Acad. Sci. U.S.A. **103**, 15812–15817 (2006). 17035506 10.1073/pnas.0510400103 PMC1595308

[51] V. Parpura, R. T. Doyle, T. A. Basarsky, E. Henderson, P. G. Haydon, Dynamic imaging of purified individual synaptic vesicles. Neuroimage **2**, 3–7 (1995). 9343585 10.1006/nimg.1995.1003

[52] D. Milovanovic, P. De Camilli, Synaptic vesicle clusters at synapses: A distinct liquid phase? Neuron **93**, 995–1002 (2017). 28279363 10.1016/j.neuron.2017.02.013 PMC5347463

[53] A. Merighi, Costorage of high molecular weight neurotransmitters in large dense core vesicles of mammalian neurons. Front. Cell Neurosci. **12**, 272 (2018). 30186121 10.3389/fncel.2018.00272 PMC6110924

[54] T. R. Zahn , Dense core vesicle dynamics in Caenorhabditis elegans neurons and the role of kinesin UNC-104. Traffic **5**, 544–559 (2004). 15180830 10.1111/j.1600-0854.2004.00195.x

[55] E. P. Karasmanis , Polarity of neuronal membrane traffic requires sorting of kinesin motor cargo during entry into dendrites by a microtubule-associated septin. Dev. Cell **46**, 518–524 (2018). 30130532 10.1016/j.devcel.2018.08.004

[56] D. P. McVicker , Transport of a kinesin-cargo pair along microtubules into dendritic spines undergoing synaptic plasticity. Nat. Commun. **7**, 12741 (2016). 27658622 10.1038/ncomms12741 PMC5411814

[57] C. I. Maeder, A. San-Miguel, E. Y. Wu, H. Lu, K. Shen, In vivo neuron-wide analysis of synaptic vesicle precursor trafficking. Traffic **15**, 273–291 (2014). 24320232 10.1111/tra.12142

[58] Q. Feng, K. J. Mickolajczyk, G. Y. Chen, W. O. Hancock, Motor reattachment kinetics play a dominant role in multimotor-driven cargo transport. Biophys. J. **114**, 400–409 (2018). 29401437 10.1016/j.bpj.2017.11.016 PMC5985011

[59] A. M. Gicking , Kinesin-1, -2, and -3 motors use family-specific mechanochemical strategies to effectively compete with dynein during bidirectional transport. eLife **11**, e82228 (2022). 36125250 10.7554/eLife.82228 PMC9545524

[60] C. Leduc , Cooperative extraction of membrane nanotubes by molecular motors. Proc. Natl. Acad. Sci. U.S.A. **101**, 17096–17101 (2004). 15569933 10.1073/pnas.0406598101 PMC535380

[61] P. Bevington, D. K. Robinson, Data Reduction and Error Analysis for the Physical Sciences (McGraw-Hill Higher Education, New York, NY, ed. 3, 2003).

[62] M. S. Woody, J. H. Lewis, M. J. Greenberg, Y. E. Goldman, E. M. Ostap, MEMLET: An easy-to-use tool for data fitting and model comparison using maximum-likelihood estimation. Biophys. J. **111**, 273–282 (2016). 27463130 10.1016/j.bpj.2016.06.019 PMC4968482

[63] S. Shastry, W. O. Hancock, Interhead tension determines processivity across diverse N-terminal kinesins. Proc. Natl. Acad. Sci. U.S.A. **108**, 16253–16258 (2011). 21911401 10.1073/pnas.1102628108 PMC3182723

[64] S. Shastry, W. O. Hancock, Neck linker length determines the degree of processivity in Kinesin-1 and Kinesin-2 motors. Curr. Biol. **20**, 939–943 (2010). 20471270 10.1016/j.cub.2010.03.065 PMC2882250

[65] J. Schindelin , Fiji: An open-source platform for biological-image analysis. Nat. Methods **9**, 676–682 (2012). 22743772 10.1038/nmeth.2019 PMC3855844

[66] G. Y. Chen, D. F. Arginteanu, W. O. Hancock, Processivity of the kinesin-2 KIF3A results from rear head gating and not front head gating. J. Biol. Chem. **290**, 10274–10294 (2015). 25657001 10.1074/jbc.M114.628032 PMC4400341

