# Supplementary material for: KIF1A is kinetically tuned to be a superengaging motor under hindering loads
Source: Proc Natl Acad Sci U S A. 2023 Jan 4;120(2):e2216903120. doi: 10.1073/pnas.2216903120 (PMC9926277; doi:10.1073/pnas.2216903120)
Supplement: Supplementary file 1 — Appendix 01 (PDF) [file pnas.2216903120.sapp.pdf]

PNAS

Supporting Information for

KIF1A is kinetically tuned to be a super-engaging motor under hindering loads

Serapion Pyrpassopoulos, Allison M. Gicking, Taylor M. Zaniewski, William O. Hancock, and E. Michael Ostap

Serapion Pyrpassopoulos
Email: serapion.pyrpassopoulos@zmbp.uni-tuebingen.de

This PDF file includes:

Supporting text
Figures S1 to S12
Tables S1 to S3
SI References

Supporting Information Text

Supplementary Methods:

Theoretical estimation of drag coefficients and relaxation times

The drag coefficient $\gamma_{microsphere}$ of a microsphere with radius $r_{microsphere}$ = 410 nm in an aqueous solution (viscosity coefficient $\eta = 10^{-9}$ pN·s/nm^2) is given by the Stokes' law (1) as $\gamma_{microsphere} = 6\pi\eta r_{microsphere} = 0.77 \times 10^{-5}$ pN·s/nm. The drag coefficient $\gamma_{dumbbell}$ of the microtubule dumbbell can be considered to a first approximation as the sum of the drag coefficients of the two beads (r = 410 nm) and that of a microtubule segment of length L = 10 μm (2). Approximating the microtubule as a solid cylinder of radius R = 12.5 nm, the drag coefficient for motion parallel to the cylindrical axis is $\gamma_{microtubule} = 2\pi\eta L/[\ln(L/(2R))-0.2]$ (1), and therefore $\gamma_{dumbbell} = 2\cdot\gamma_{microsphere} + \gamma_{microtubule} = 2.7 \times 10^{-5}$ pN·s/nm = $3.5\cdot\gamma_{microsphere}$. Since the characteristic relaxation time of a laser trapped object is $\tau = \gamma/k$, where k is the stiffness of the laser trap, the microtubule dumbbell will have similar τ to that of a microsphere only if trap stiffness k~0.04 pN/nm is increased by the same factor as the drag coefficient, i.e 3.5 x 0.04 pN/nm = 0.14 pN/nm.

Calculation speed as a function of force

To calculate the profile of speed as a function of force two different but equivalent approaches were used:

1. The average of the displacement ramps as a function of time was calculated as previously described (3), and was smoothed using a Savitzky-Golay filter with a 20-point window. The speed was calculated from the first derivative of the smoothed average displacement trace. The corresponding force value was calculated by multiplication of the average displacement value with the optical trap stiffness.
2. The speed was calculated for each displacement ramp by linear fit of successive 10 ms window segments. The corresponding force value was calculated by the average displacement value of each segment multiplied by the optical trap stiffness.

Both methods gave similar results as can be seen is Fig. S10.

Subtilisin treatment of polymerized microtubules

We adopted a modified version of the protocol from Rodionov et al. (4). Taxol-stabilized GDP microtubules (50 μM tubulin) were incubated with 1 μM of A subtilisin for 4 hours at 37 °C water bath. The reaction was then blocked by the addition of PMSF to final concentration of 2 mM and the sample was kept at RT for at least 1 hour before using it for further experiments. We confirmed by western blot that for shorter incubation periods the C-terminal tail of α-tubulin is not fully cleaved as has been reported previously (5) (Fig. S8). Primary antibodies specific for the C-terminal tail of α and β tubulin were used (see Materials and Methods).

Ionic Strength Calculations

The two buffers used in this study were BRB80 (80 mM PIPES, 1 mM $MgCl_2$, 1mM EGTA, 2 mM MgATP, pH 6.9) and BRB12 (12 mM PIPES, 1 mM $MgCl_2$, 2 mM MgATP, 1 mM EGTA, pH 6.9). The PIPES is titrated to pH 6.9 with KOH, and 2 mM MgATP is formulated by combining 2 mM $MgCl_2$ with 2 mM disodium ATP (thus adding an additional 2 mM NaCl). The ionic strength is calculated as (6):

$$I = \frac{1}{2}\sum c_i z_i^2$$

PIPES has two ionizable protons, with PIPES to $PIPES^{-1}$ having a pKa of 2.67 and $PIPES^{-1}$ to $PIPES^{-2}$ having a pKa of 6.76 (7). Thus, whether PIPES is formulated from the protonated form, or by combining mono- and dibasic forms, it will include slightly more than 1.5 molar equivalents of K^+. The Henderson-Hasselbalch equation is:

$$pH = pK_a + log_{10}\left(\frac{[Base]}{[Acid]}\right)$$

Here $PIPES^{-1}$ is the acid form and $PIPES^{-2}$ is the basic form. The equation can be rearranged to calculate the fraction of PIPES in the acid form as:

$$\frac{[Acid]}{[Acid]+[Base]}=\frac{1}{1+10^{(pH-pK_a)}}$$

Thus, the contribution of PIPES to the ionic strength at pH 6.9 is:

$[PIPES]_{total}$	$[PIPES^{-1}]$	$[PIPES^{-2}]$	$[K^+]$	Ionic Strength
80 mM	33.6 mM	46.4 mM	126.4 mM	173 mM
12 mM	5.0 mM	7.0 mM	19.0 mM	26 mM

The other three components are Mg^{2+}; EGTA, a Ca^{2+} chelator that binds Mg^{2+} weakly; and ATP, which binds Mg^{2+} with a dissociation of 50 µM (8). The Mg^{2+} binding affinities of EGTA and ATP, as well as the charge of these species all vary with temperature, pH, and ionic strength, requiring the use of a buffer calculator. We used Maxchelator, developed by Chris Patton, which is described fully in (9) and is online at: https://somapp.ucdmc.ucdavis.edu/pharmacology/bers/maxchelator/index.html. The program "Ca-Mg-ATP-EGTA Calculator v1.0 using constants from NIST database #46 v8" was used to calculate the free and bound species, and their contribution to the overall ionic strength of the buffer, as follows:

**BRB80** (20 °C, pH 6.9, estimated ionic strength 185 mM)			
**Component**	**Total Conc.**	**Free Conc.**	**Bound Conc.**
Mg^{2+}	3.0 mM	1.22 mM	1.78 mM
ATP	2.0 mM	0.24 mM	1.76 mM
EGTA	1.0 mM	0.97 mM	0.03 mM
**Ionic strength contribution = 5.8 mM**			

**BRB12 (**20 °C, pH 6.9, estimated ionic strength 35 mM)			
**Component**	**Total Conc.**	**Free Conc.**	**Bound Conc.**
Mg^{2+}	3.0 mM	1.05 mM	1.95 mM
ATP	2.0 mM	0.09 mM	1.91 mM
EGTA	1.0 mM	0.96 mM	0.04 mM
**Ionic strength contribution = 5.2 mM**			

Combining the contributions of PIPES, Mg/ATP/EGTA, and the 4 mM Na^+ from the disodium ATP and 6 mM Cl^- from the $MgCl_2$, the final estimated ionic strengths are as follows:

**Buffer**	**Ionic Strength Contribution**				**Total Ionic Strength**
	**PIPES + K^+**	**Mg/ATP/EGTA**	**Na^+**	**Cl^-**	
**BRB80**	173 mM	5.8 mM	2 mM	3 mM	**184 mM**
**BRB12**	26 mM	5.2 mM	2 mM	3 mM	**36 mM**

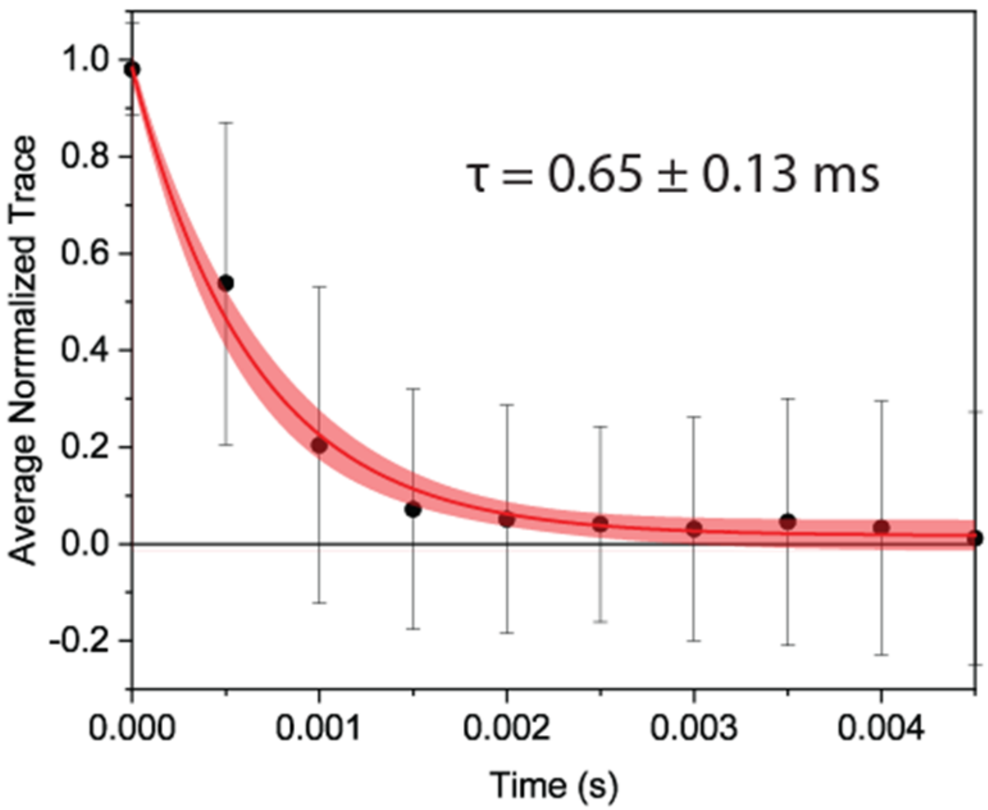

Fig. S1. **Average relaxation trace for 3-Bead assay.**
Relaxation traces from one dumbbell that correspond to either primary or secondary events and were strictly monotonic within 5 ms were normalized and then averaged (scatter points). Error bars correspond to standard deviations. The red line corresponds to single-exponential curve fitting and the 95% confidence band is indicated by the lighter red color.

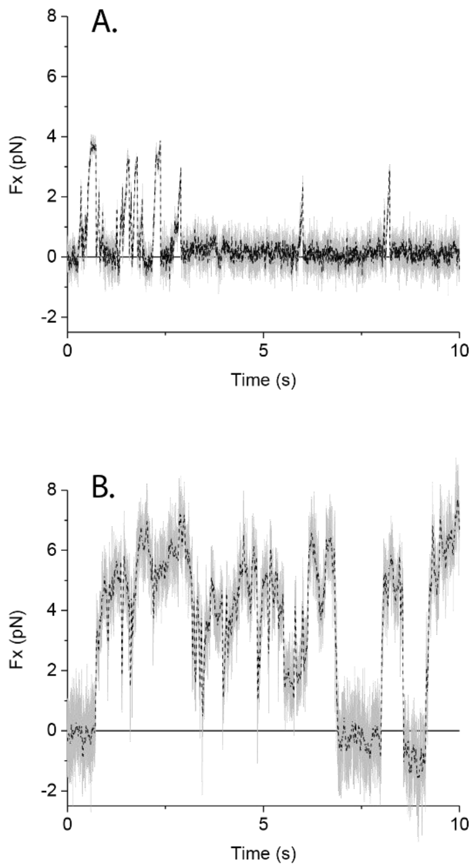

Fig. S2. Force traces of KIF1A for 1-Bead and 3-Bead assays.
Force traces of KIF1A from Fig. 1D & E are presented in a more expanded time scale of 10 s.

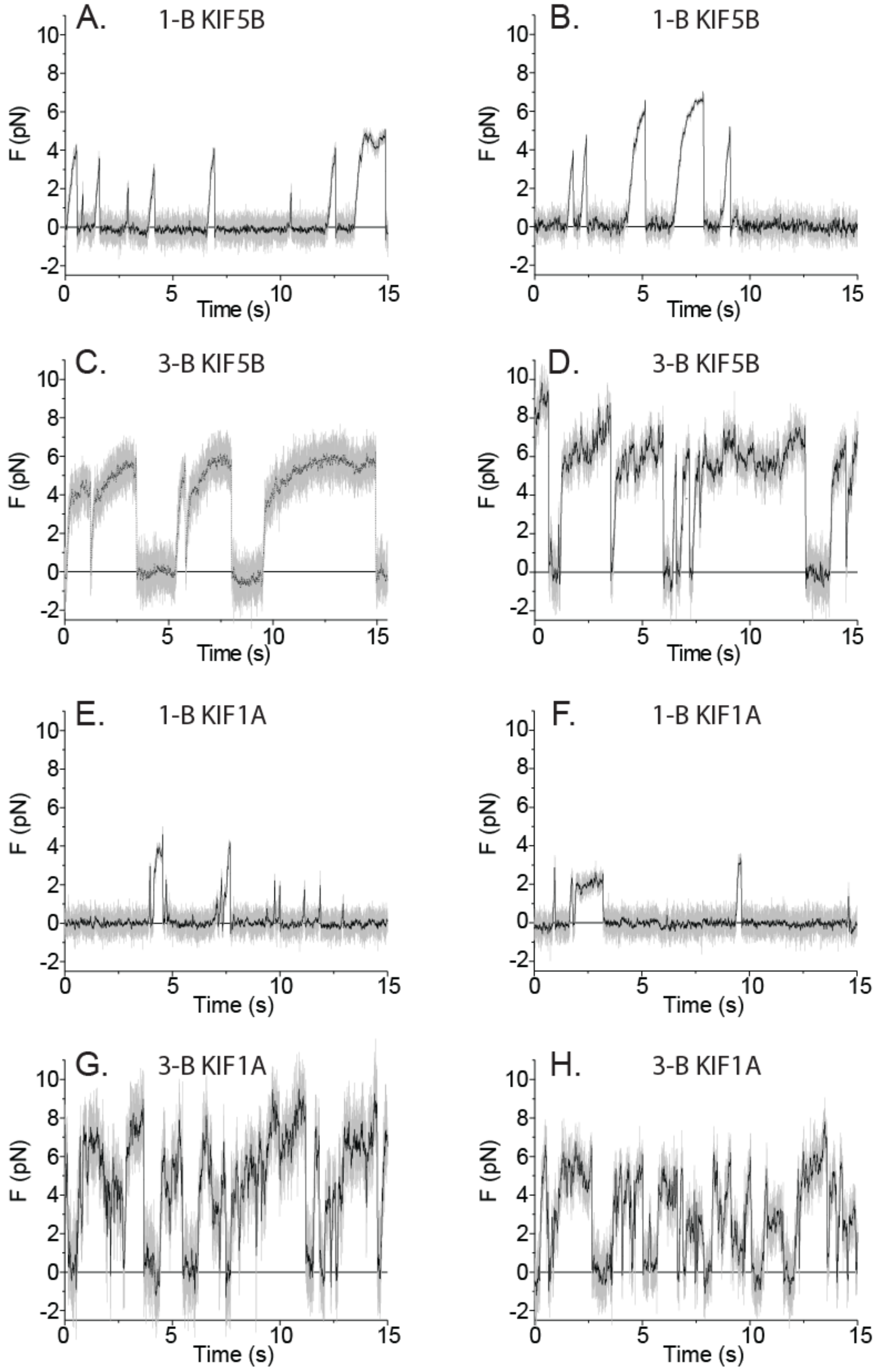

Fig. S3. **Gallery of force trace of KIF5B and KIF1A for 1-Bead and 3-Bead assays.**
Force traces of KIF5B for the single-bead assay in panels (A) and (B), and for the three-bead assay in panels (C) and (D). Force traces of KIF1A for the single-bead assay in panels (E) and (F), and for the three-bead assay in panels (G) and (H).

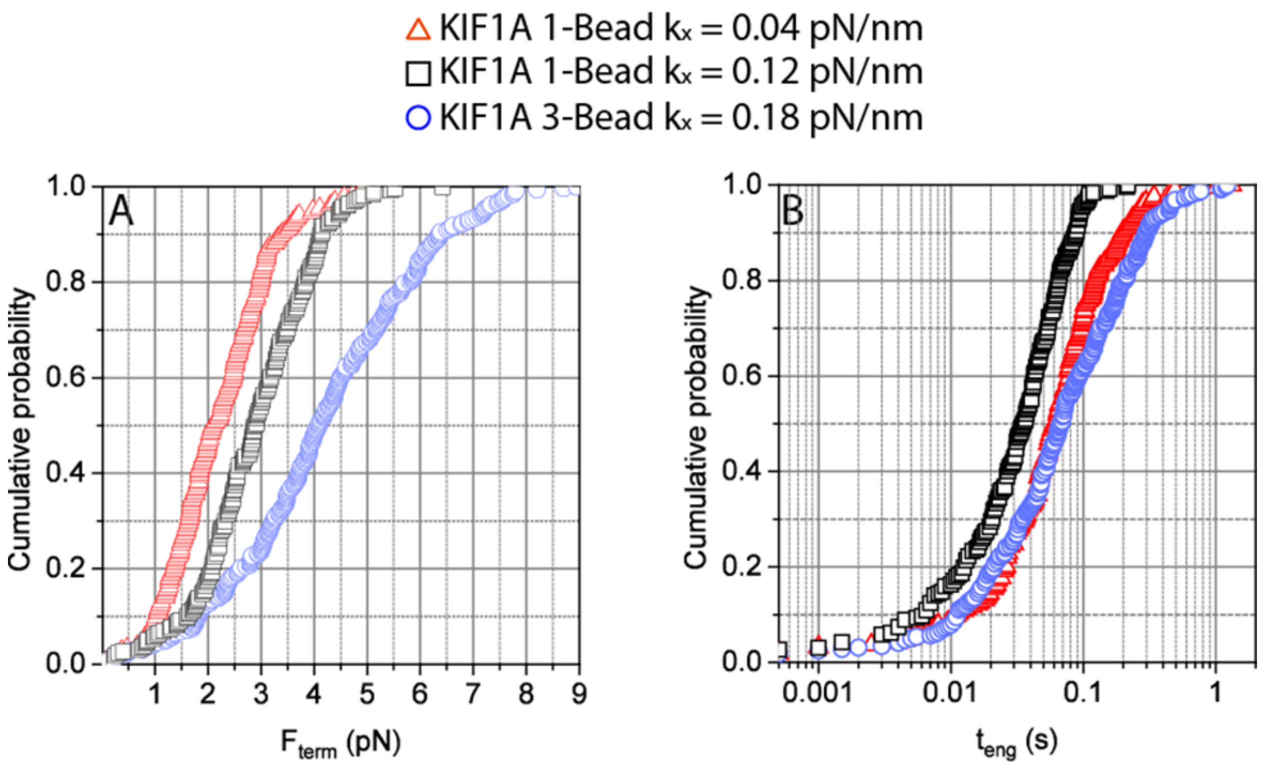

Fig. S4. Comparison of 1-bead data at higher and lower stiffness, with the 3-bead assay. Cumulative probability distributions of terminal forces (A) and force ramp durations (B) for the single-bead and three-bead assays. To test whether larger the F_{term} in the three-bead assay (blue, open circles) was due to the higher trap stiffness, the trap stiffness was tripled in the single-bead assay from 0.04 pN/nm (red, open upright triangle) to 0.12 pN/nm (black, open square). Although there was a moderate shift toward the three-bead values, F_{term} values are still clearly smaller in the one-bead assay. For t_{eng}, increasing the single-bead trap stiffness closer to the three-bead value shortened t_{eng}, reflecting the faster dissociation rates under load in the single-bead assay.

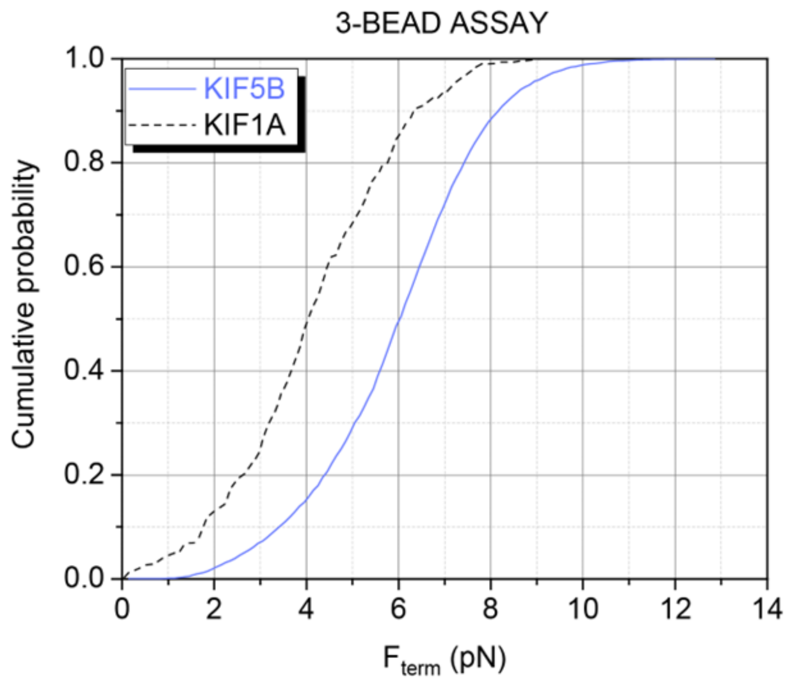

Fig. S5. **Cumulative probability of F_{term} for KIF1A and KIF5B in the three-bead assay.**

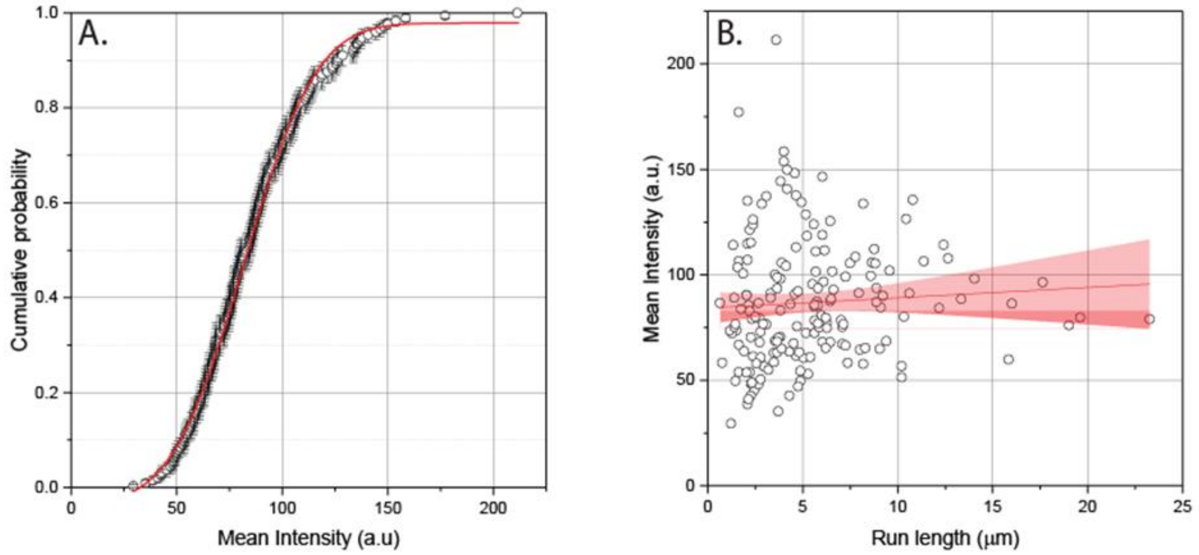

Fig. S6. **Fluorescence intensity distribution from TIRF single-molecule events and its correlation with the run length**.

(A) Cumulative probability of the fluorescence intensity from single-molecule events observed by TIRF. Error bars were calculated by the bootstrap method. The red line represents a weighted fit to cumulative distribution function of the standard normal distribution. (B) Plot of the mean fluorescence intensity of single-molecule events as a function of the corresponding running length. The red line represents linear fit and the red band the 95% confidence interval of the fit. The slope was not significantly different than zero at 0.05 significance level.

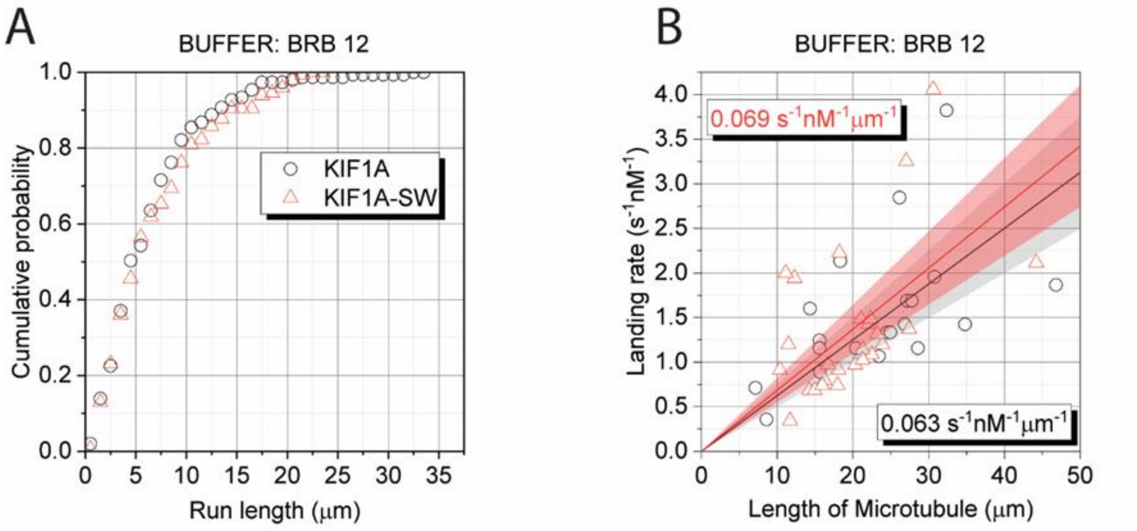

Fig. S7. Run length and landing rate of KIF1A and KIF1A-SW in buffer BRB12.
(A) Cumulative probability of the run length and (B) landing rates for single KIF1A (black) and KIF1A-SW (red) molecules observed under TIRF microscopy in BRB12 buffer.

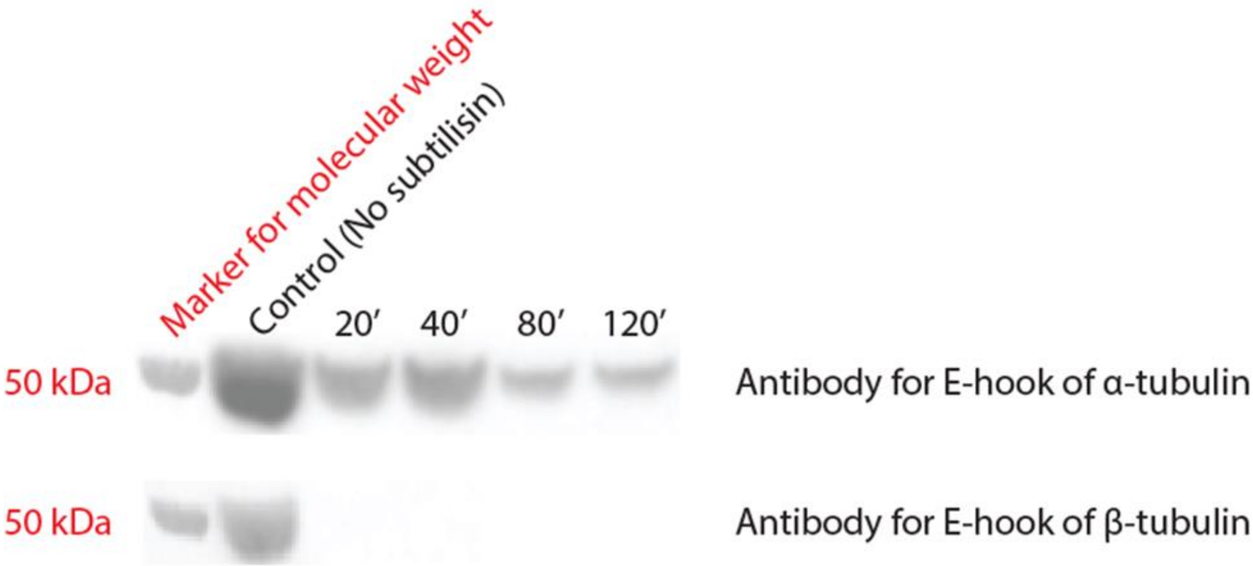

Fig. S8. **Time-course of subtilisin mediated cleavage of tubulin's C-terminal tails from polymerized microtubules stabilized with Taxol.**

Western-blot against the C-termini of α and β tubulin after treatment of polymerized microtubules with subtilisin at 37 °C for 20, 40, 80 and 120 min (see Supplemental Materials and Methods).

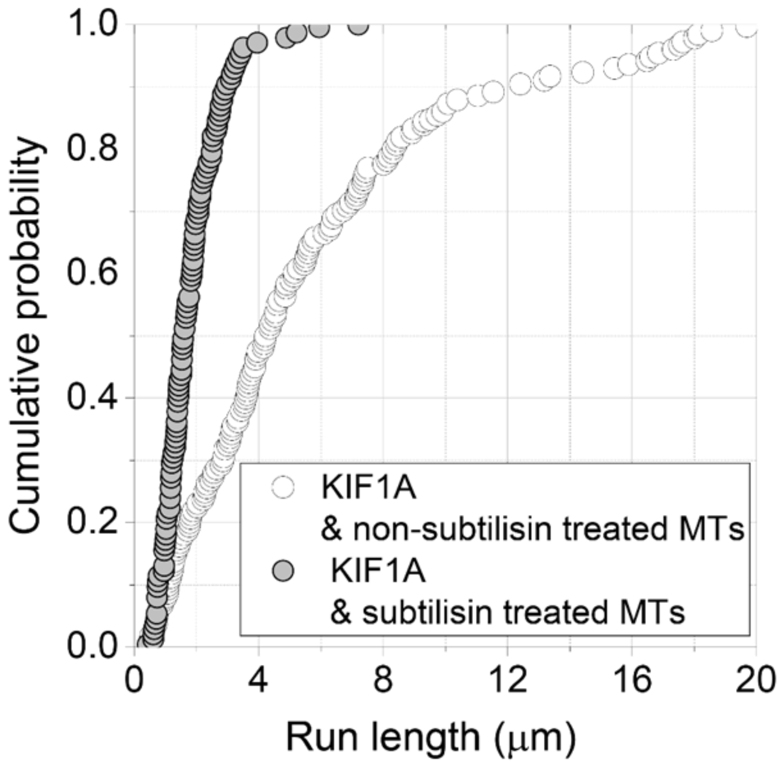

Fig. S9. **Unloaded run length of KIF1A on MTs that were treated and not with subtilisin.** Cumulative probability of the run length for single KIF1A molecules observed under TIRF microscopy in BRB 80 buffer when microtubules were treated with subtilisin (gray filled circle) and not (open black circles).

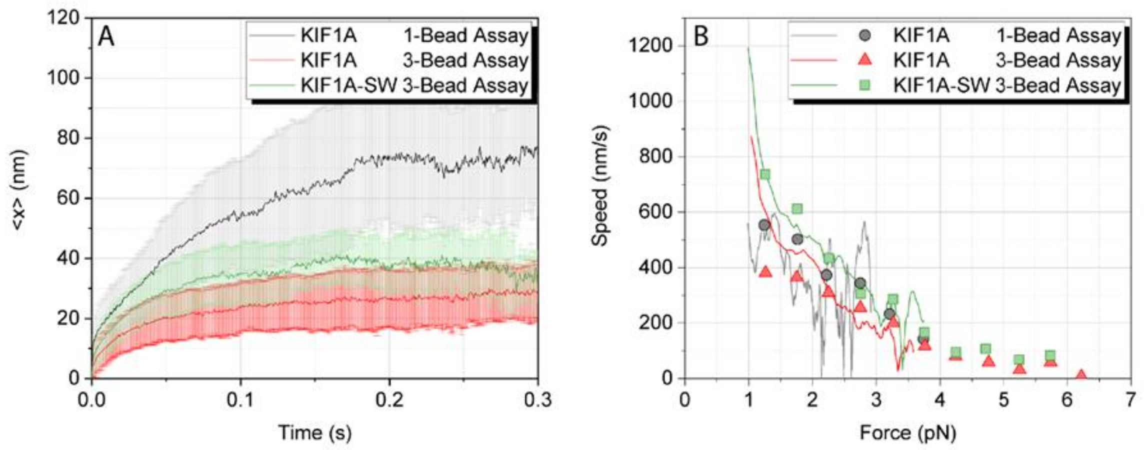

Fig. S10. **Ensemble trajectories and force-velocity curve.**
(A) Ensemble average of displacement ramps for single- and three-bead assays and KIF1A constructs using only the primary events. (B) Speed as a function of force calculated either from the derivative of the ensemble average trace (continuous line) or by piecewise calculation of the velocity for each displacement ramp over a 10 ms time window (not sliding) and subsequent averaging over all displacement ramps for each assay and KIF1A construct (scatter points), as described in Supplemental materials and methods.

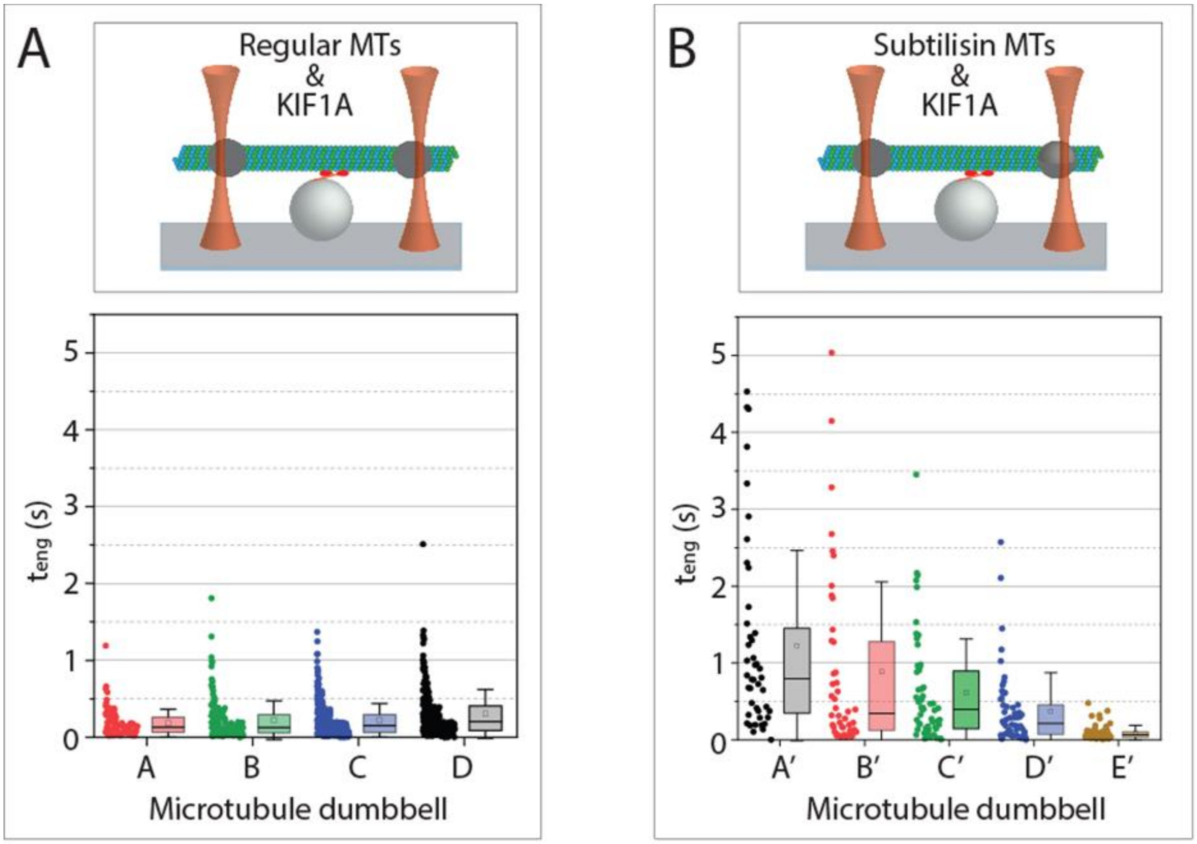

Fig. S11. **Three-bead assay using subtilisin treated microtubules.**
Statistics-box plot of the attachment durations t_{eng} between KIF1A single-molecules and (A) microtubule dumbbells not treated with subtilisin and (B) microtubule dumbbells after treatment with subtilisin A (see Supplemental Materials and Methods).

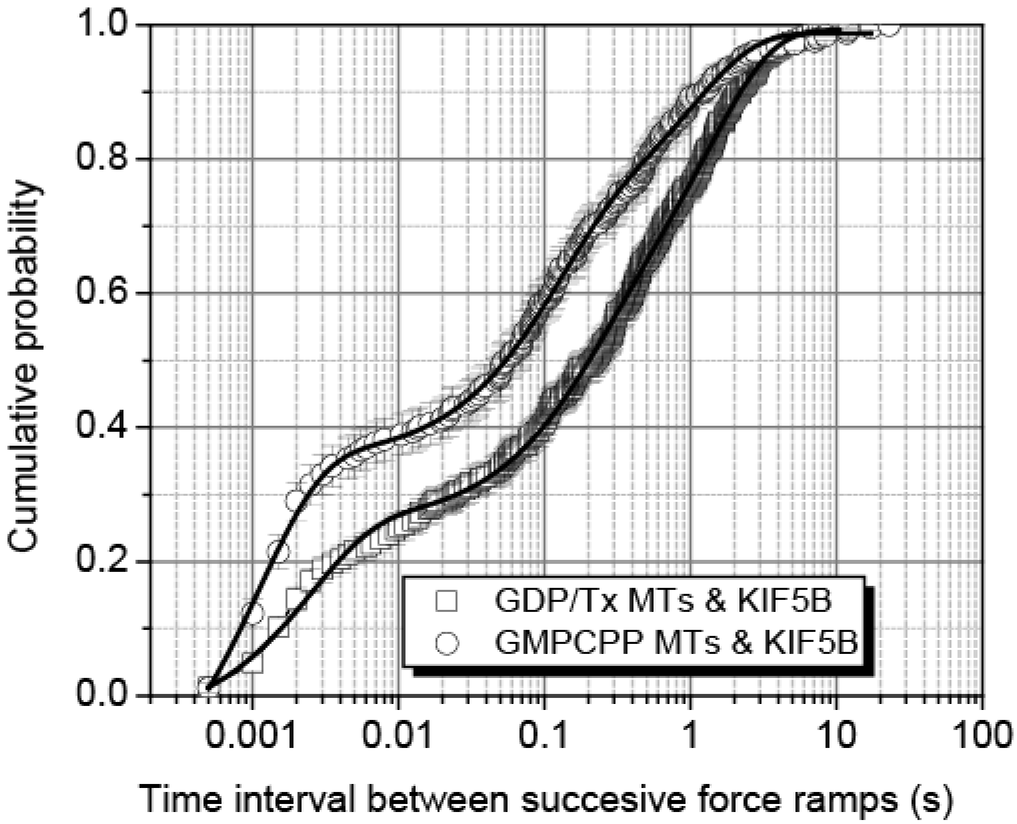

Fig. S12. **Comparison of KIF5B restart times on different microtubules.**
Cumulative probability plot of the time intervals between successive force ramps for K560 on taxol (open squares) and GMPCCP (open circles) stabilized microtubules, using the three-bead assay. Error bars are calculated using the bootstrap method (11) and the solid lines represent fitting to three-exponential decay function (see Materials and Methods).
Table S1. Type or paste table title here. Paste table below the title.

Table S1.

Assay	Construct	% Secondary Events	% Secondary Events
		**Taxol/GDP MTs**	**GMPCPP MTs**
Single-bead	KIF1A	11	-
	KIF5B	10	-
Three-bead	KIF1A	39	44
	KIF5B	13	22
	KIF1A-SW	26	49

Table S2. Type or paste table title here. Paste table below the title.

Assay	Motor	**Primary Events Taxol/GDP MTs**		**Secondary Events Taxol/GDP MTs**		**Primary Events GMPCPP MTs**		**Secondary Events GMPCPP MTs**	
		$\langle F_{term} \rangle^{(a)}$ (pN)	Med-t_{eng} (s)	$\langle F_{term} \rangle^{(a)}$ (pN)	Med- t_{eng} (s)	$\langle F_{term} \rangle^{(a)}$ (pN)	Med- t_{eng} (s)	$\langle F_{term} \rangle^{(a)}$ (pN)	Med- t_{eng} (s)
1-bead	KIF1A	2.2 ± 0.99	0.062	2.6 ± 0.77	0.023	-	-		
3-bead	KIF1A	4.1 ± 1.8	0.069	5.0 ± 0.11	0.067	3.2 ± 1.3	0.065	4.3 ± 1.5	0.079
	KIF1A-SW	3.5 ± 1.4	0.039	4.7 ± 1.4	0.030	3.4 ± 1.5	0.033	4.9 ± 1.6	0.043

[a] Since the distribution of termination forces are symmetrical (Fig 2D, F), the $\langle F_{tepm} \rangle$ is almost identical with the median value of F_{term}.

Table S3.

Construct	<Speed> (μm/s)	<Run Length> (μm)	Med- t_{eng} (s)
KIF1A	1.1 ± 0.38	6.3 ± 4.2(*)	3.75
KIF1A-SW	1.3 ± 0.42	1.1 ± 0.56	0.73
KIF1A & Subtilisin MTs	1.3 ± 0.39	1.8 ± 1.1	1.3

(*) The value has been corrected for events in which the motor runs all the way to the end of the microtubule (10).

SI References

1. J. Howard, *Mechanics of Motor Proteins and the Cytoskeleton* (Sinauer Associates, Inc., Sunderland, MA, ed. 1st., 2001), pp. 367.
2. S. Pyrpassopoulos *et al.*, Force Generation by Membrane-Associated Myosin-I. *Sci Rep* **6**, 25524 (2016).
3. S. Pyrpassopoulos, H. Shuman, E. M. Ostap, Modulation of Kinesin's Load-Bearing Capacity by Force Geometry and the Microtubule Track. *Biophys J* **118**, 243-253 (2020).
4. V. I. Rodionov, F. K. Gyoeva, A. S. Kashina, S. A. Kuznetsov, V. I. Gelfand, Microtubule-associated proteins and microtubule-based translocators have different binding sites on tubulin molecule. *J Biol Chem* **265**, 5702-5707 (1990).
5. B. M. Paschal, R. A. Obar, R. B. Vallee, Interaction of brain cytoplasmic dynein and MAP2 with a common sequence at the C terminus of tubulin. *Nature* **342**, 569-572 (1989).
6. P. W. Atkins, *Physical Chemistry* (Oxford University Press, Oxford, UK, ed. Third, 1986).
7. N. E. Good *et al.*, Hydrogen ion buffers for biological research. *Biochemistry* **5**, 467-477 (1966).
8. R. K. Gupta, P. Gupta, W. D. Yushok, Z. B. Rose, Measurement of the dissociation constant of MgATP at physiological nucleotide levels by a combination of 31P NMR and optical absorbance spectroscopy. *Biochem Biophys Res Commun* **117**, 210-216 (1983).
9. D. M. Bers, C. W. Patton, R. Nuccitelli, A practical guide to the preparation of Ca(2+) buffers. *Methods Cell Biol* **99**, 1-26 (2010).
10. T. M. Zaniewski, A. M. Gicking, J. Fricks, W. O. Hancock, A kinetic dissection of the fast and superprocessive kinesin-3 KIF1A reveals a predominant one-head-bound state during its chemomechanical cycle. *J Biol Chem* **295**, 17889-17903 (2020).
11. W. H. Presse, P. B. Flannery, S. A. Teukolsky, W. T. Vetterling, *Numerical Recipes in C: The Art of Scientific Computing 2nd Ed.* (Cambridge University Press, Cambridge, U.K., 1988).
